# Biophysics-informed computational framework for predicting antisense oligonucleotide knockdown efficacy under small-data constraints

**DOI:** 10.3389/fsysb.2026.1921636

**Published:** 2026-10-14

**Authors:** Risha Guha, Shawn Kim, Anwita Bandaru, Nithika Vivek

**Affiliations:** Del Norte High School, San Diego, CA, United States

**Keywords:** amyotrophic lateral sclerosis, antisense oligonucleotide, knockdown, machine learning, protein interactions

## Abstract

A central strategy in treating neurodegenerative disorders, including amyotrophic lateral sclerosis (ALS), is lowering levels of disease-driving proteins. Antisense oligonucleotides (ASOs) offer a direct RNA-targeted approach to reduce protein expression, but experimental discovery and optimization are constrained by small, heterogeneous datasets and target-specific variability. Computational models that predict ASO knockdown efficacy directly from sequence and context could improve candidate selection, yet generalization across gene families and experimental settings remains challenging. In this study, we present a biophysics-based computational framework for predicting RNase H-active ASO knockdown efficacy under small-data constraints. We curate and integrate heterogeneous literature-derived ASO efficacy measurements into a unified dataset and evaluate model transfer using independently generated experimental ASOs targeting ALS-relevant genes DAZAP1 and FAM69C. Knockdown efficacy is defined at the transcript level (%KD), providing a mechanistic measure of RNase H-mediated activity. To our knowledge, this is the first framework that combines ASO feature engineering with one-shot family calibration for prediction under realistic small-data conditions. We integrate sequence representations with physics-based features capturing RNA-DNA hybridization thermodynamics, targeting RNA accessibility. Compared to sequence-only baselines, this mechanistic feature augmentation reduces external wet-lab prediction error, improving generalization to independent experimental data. Despite these gains, uncalibrated predictions exhibit systematic target-family-specific scale bias. We address this challenge by using a minimal one-shot family calibration strategy that applies an additive offset derived from a single experimentally measured reference ASO per new target family. We further assess the model’s behavior using a leave-one-family-out simulation on literature-derived ASO families, repeatedly applying one-shot calibration to quantify its expected benefit and variability under controlled small-n conditions. One-shot calibration markedly improves absolute prediction accuracy while preserving within-family ranking, without retraining or increasing model complexity. Out-of-distribution evaluation shows stable performance for DAZAP1 and increased uncertainty for FAM69C, consistent with the expected effects of very small sample sizes. Permutation-based interpretability analyses show that predictive signal is dominated by global sequence architecture, with thermodynamic and accessibility features providing correlated, secondary contributions. Together, these results establish a computational workflow in which literature-trained models can be transferred to new target genes using minimal experimental input, supporting systematic ASO prioritization in small-data models.

## 1 Introduction

In amyotrophic lateral sclerosis (ALS), aggregation and mislocalization of TAR DNA-binding protein 43 (TDP-43) are closely linked to disease progression and neuronal toxicity. TDP-43 pathology is strongly associated with stress-granule biology: stress granules frequently co-localize with TDP-43 inclusions, and chronic cellular stress promotes aberrant stress-granule persistence and TDP-43 redistribution (Dewey et al., 2012; Ding et al., 2021; Jo et al., 2020). Antisense oligonucleotides (ASOs) provide a direct RNA-targeted approach to mitigate these processes by reducing target expression through RNase H-mediated cleavage of RNA-DNA heteroduplexes or, in some designs, steric interference with translation or splicing (Shen and Corey, 2018; Crooke et al., 2021; Dhuri et al., 2020). RNase H-active gapmer ASOs are critical for neurological drug development, with multiple ALS-relevant targets advancing from preclinical studies to clinical trials (Schoch and Miller, 2017; Bennett et al., 2021; Roberts et al., 2022; Miller et al., 2026). The principal molecular mechanisms by which ASOs modulate gene expression are summarized in Figure 1.

**FIGURE 1:**
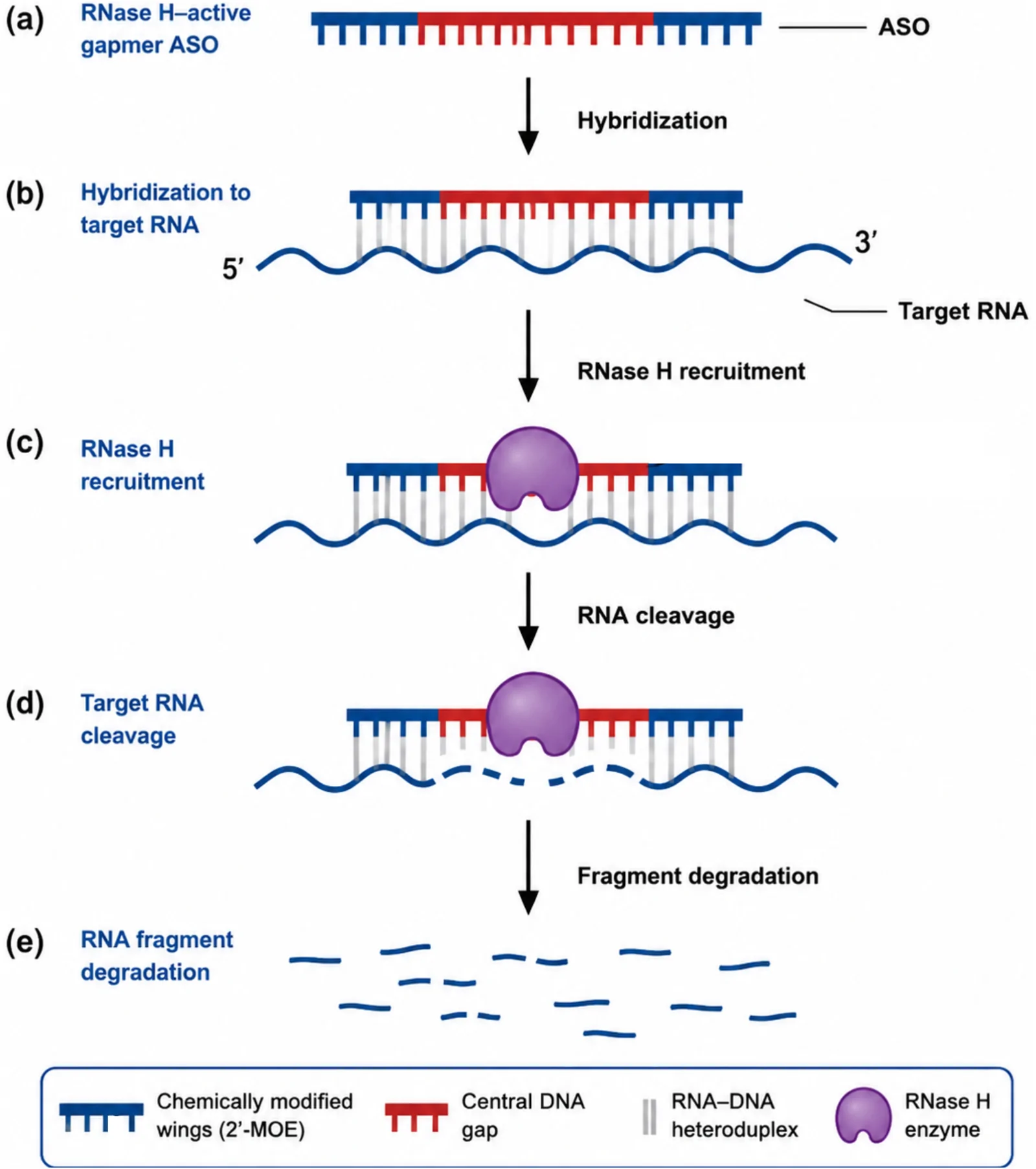
Mechanism of RNase H-mediated knockdown by a gapmer ASO. **(a)** A gapmer ASO, consisting of a central DNA gap flanked by chemically modified wings, is designed to bind a complementary target RNA sequence. **(b)** Hybridization of the ASO with its target RNA forms an RNA–DNA heteroduplex. **(c)** The heteroduplex recruits RNase H, which specifically recognizes RNA–DNA duplexes. **(d)** RNase H cleaves the target RNA while leaving the ASO intact, allowing the ASO to dissociate and participate in additional rounds of target recognition. **(e)** The cleaved RNA fragments are subsequently degraded, resulting in reduced target transcript abundance (knockdown efficacy).

Despite their therapeutic promise, ASO discovery and optimization remain experimentally intensive. To knock down a single protein, dozens to hundreds of candidate 20-mer windows may be considered, each compatible with multiple chemical architectures, backbone modifications, and assay contexts. Exhaustive wet-lab screening across this combinatorial design space is costly and time-consuming, limiting both the number of variants tested per target and the number of targets explored overall. Computational approaches that estimate knockdown efficacy directly from ASO sequence and context offer a means to prioritize candidates for experimental testing and reduce development cost (Chalk and Sonnhammer, 2002; Sciabola et al., 2021; Hwang et al., 2024). However, ASO efficacy modeling is currently constrained by the nature of available evidence: datasets are typically small, heterogeneous, and aggregated from diverse sources, with labels reported as exact percentages, ranges, or qualitative categories, and with inconsistent annotation of chemistry, assay conditions, and sequence context (Rigo et al., 2014; Yoshida et al., 2019; Hagedorn et al., 2022). Because of this, several challenges persist, including limited data per gene family, systematic shifts across targets and assay systems, and the frequent disconnect between chemically favorable ASO designs and regions of the target RNA that are structurally accessible for productive binding (Yoshida et al., 2019; Hagedorn et al., 2022; Hörberg et al., 2024).

Prior computational efforts have addressed ASO and siRNA activity prediction using progressively more sophisticated strategies. Early models relied on rule-based heuristics and thermodynamic proxies derived from sequence composition, GC content, and nearest-neighbor energetics (Sugimoto et al., 1995; Chalk and Sonnhammer, 2002; SantaLucia and Hicks, 2004). Subsequent approaches incorporated linear and kernel-based regression, neural networks, and, more recently, deep-learning architectures trained on sequence and chemical representations. Several computational platforms have recently been developed to support different stages of ASO design and optimization. Contemporary examples include PFRED, an open-source platform that integrates transcript annotation, candidate enumeration, sequence filtering, structural analysis, and off-target assessment for RNase H-dependent ASOs; eSkip-Finder which combines sequence, binding-energy, accessibility, and splice-context features to predict exon-skipping efficacy; ASOptimizer which uses deep learning to optimize both ASO sequence and chemical modification patterns; and OligoGym which provides standardized oligonucleotide datasets and benchmarks for classical and deep-learning models (Sciabola et al., 2021; Chiba et al., 2021; Hwang et al., 2024; Rotrattanadumrong and De Donno, 2025). In parallel, advances in ASO chemistry and delivery have improved stability, selectivity, and clinical feasibility, particularly for RNase H-dependent gapmers used in neurological indications (Kamola et al., 2015; Schoch and Miller, 2017; Dhuri et al., 2020; Liu et al., 2025).

Collectively, these studies have established the value of computational methods for ASO design; however, an important translational gap remains. Candidate-generation platforms such as PFRED primarily enumerate and rank potential ASOs, whereas eSkip-Finder models steric-blocking exon skipping rather than RNase H-mediated transcript degradation. ASOptimizer and related deep-learning approaches benefit from substantially larger aggregated datasets, but they generally do not address the practical problem encountered when a newly investigated gene has few or no target-specific efficacy measurements. Differences in transcript structure, chemistry, delivery, cell system, and assay context can produce systematic shifts in efficacy between target families, limiting transfer from a fixed global model. Consequently, a complementary need remains for computational approaches that operate under heterogeneous, small-data conditions and can adapt predictions to previously unseen RNase H targets using minimal experimental information.

We focused on ALS and neurodegeneration as a biologically coherent test domain because RNase H-active gapmers are clinically established in neurological disease, while publicly accessible sequence-resolved efficacy measurements remain limited and heterogeneous across individual targets. Restricting the study to ASOs with comparable mechanisms, molecular endpoints, and design classes reduced confounding that would arise from combining unrelated diseases, chemistries, delivery systems, and modes of action. The resulting framework should therefore be interpreted as a proof of concept for data-limited cross-target transfer rather than a disease-comprehensive or universally trained ASO model. It integrates literature-derived efficacy measurements with independent ASOs targeting previously unseen genes and introduces one-shot family calibration, in which a single measured ASO corrects target-specific prediction bias without model retraining or alteration of within-target ranking. This approach directly addresses the gap between globally trained predictors and the experimentally constrained development of ASOs for new or understudied targets.

Standardized sequences were used to compute biologically motivated features capturing sequence composition, gapmer structure, and RNA–DNA hybridization properties. Where available, RNA secondary-structure accessibility estimates were incorporated to reflect target-site availability, as illustrated in Figure 2. Predictive models were trained using a gradient-boosting regression approach designed for small, family-structured datasets, with an additional family-aware calibration step applied when transferring to new target genes.

**FIGURE 2:**
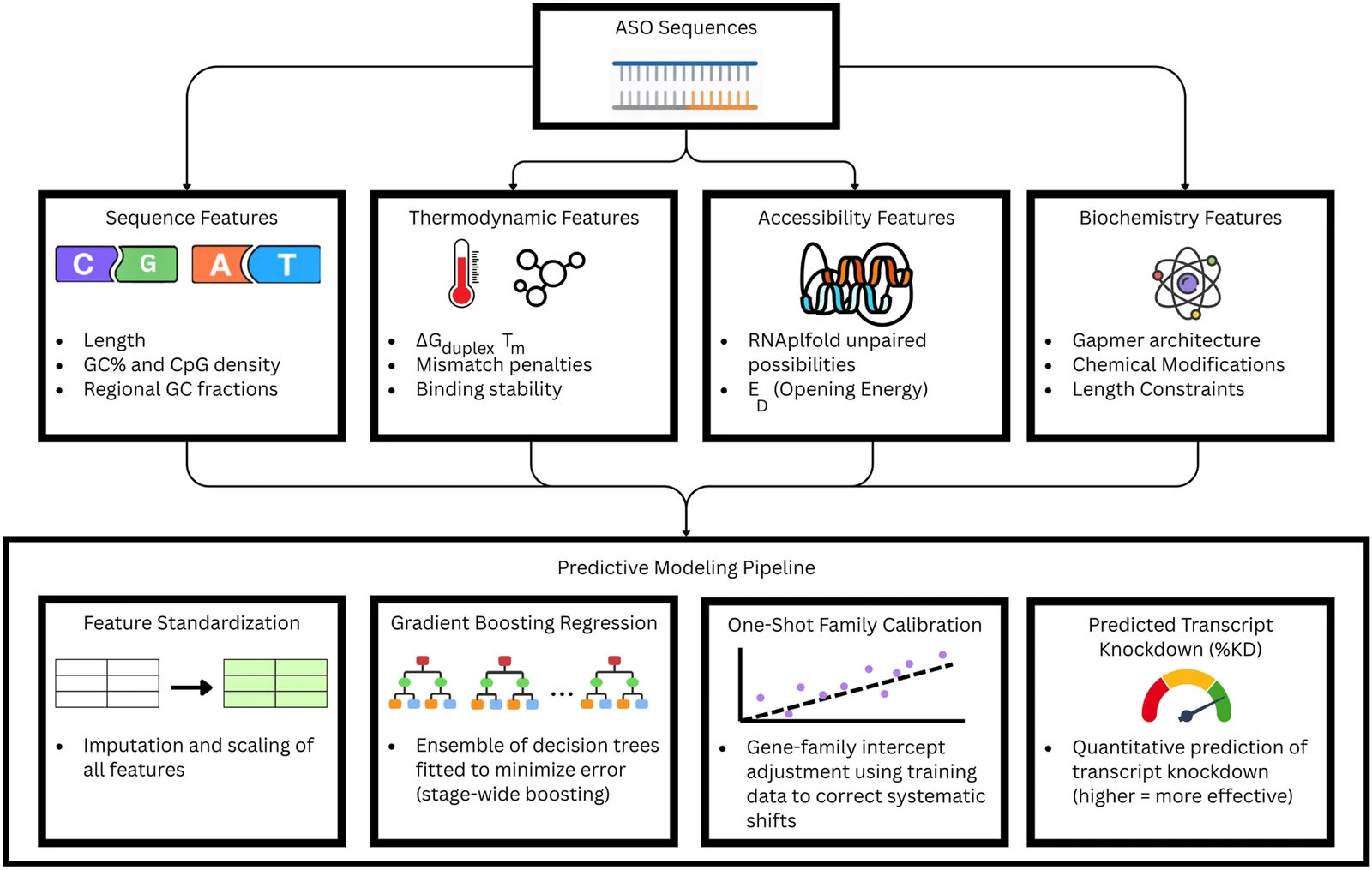
Overview of the feature engineering and predictive modeling framework used for ASO knockdown prediction. ASO sequences are transformed into four biologically motivated feature classes: sequence features, including nucleotide composition and regional GC content; thermodynamic features, including RNA–DNA duplex stability and hybridization energetics; RNA accessibility features, including RNAplfold-derived accessibility descriptors and opening energy; and biochemical features, representing gapmer architecture, chemical modifications, and design constraints. Following feature engineering, all descriptors are standardized and supplied to a gradient boosting regression model, which learns non-linear relationships between engineered features and transcript knockdown efficacy through an ensemble of shallow decision trees. Model predictions are subsequently refined using one-shot family calibration to correct systematic target-family-specific prediction offsets before generating the final quantitative prediction of transcript %KD. The dashed box denotes the predictive modeling pipeline applied to all engineered feature sets.

Model performance was evaluated using family-aware cross-validation, leave-one-family-out testing, and true out-of-distribution assessment on experimentally measured targets. Evaluation metrics and confidence intervals were pre-specified, and all analyses were conducted using fixed data partitions and versioned feature artifacts to ensure reproducibility.

## 2 Methods

### 2.1 Overview of study design

Our analytical framework consists of three phases: data harmonization, biophysical feature engineering, and predictive modeling with family-aware calibration. First, heterogeneous literature-derived and in-house experimental ASO efficacy measurements were standardized into a unified schema, establishing transcript-level %KD as the primary quantitative endpoint. Second, ASO sequences were mapped to a compact feature space that couples sequence-level composition with biophysical proxies for RNA-DNA duplex hybridization energetics and target RNA accessibility (SantaLucia and Hicks, 2004; Mathews and Turner, 2006). Finally, predictive modeling was executed using a shallow gradient-boosting architecture appropriate for small-n, family-structured datasets (Friedman, 2001; Chen and Guestrin, 2016). To correct systematic inter-family bias, models were augmented with an additive, one-shot calibration step utilizing a single reference ASO per target family, avoiding the instability of monotonic re-mapping in very small samples. Generalization was subsequently evaluated via family-aware cross-validation and true out-of-distribution testing on independent wet-lab data.

ASO efficacy data from published literature and independent experimental studies were consolidated into a unified schema. Reported knockdown labels were harmonized across heterogeneous formats and subjected to quality control prior to feature computation. A biologically grounded feature panel capturing sequence composition, gapmer architecture, RNA-DNA hybridization energetics, and target RNA accessibility was generated and cached as a versioned artifact. This standardized feature representation served as the fixed input for all subsequent model training, calibration, and evaluation steps.

### 2.2 Data sources and study scope

This study integrates heterogeneous ASO efficacy data derived from both published literature and independently generated experimental measurements. All datasets were restricted to ASOs designed to modulate gene expression through RNA binding, with primary emphasis on RNase H-active gapmer architectures, which remain central to neurological and ALS-associated therapeutic development (Schoch and Miller, 2017; Shen and Corey, 2018; Bennett et al., 2021).

Literature-derived data were collected from peer-reviewed studies reporting ASO activity against ALS-relevant genes, including canonical disease targets such as SOD1 and ATXN2, as well as related stress-granule-associated pathways implicated in TDP-43 pathology (Lagier-Tourenne et al., 2012; Korben et al., 2022; Miller et al., 2026). Reported efficacy labels varied substantially across sources and included exact percentage knockdown values, bounded ranges, and qualitative descriptors (e.g., high, medium, low), reflecting differences in assay systems and reporting practices common in the ASO literature. Where available, associated metadata such as ASO sequence, target gene, chemical design, assay modality, and reference provenance were retained. The initial literature aggregation comprised 150 efficacy records extracted from peer-reviewed publications and patent disclosures and served as a source-level curation resource rather than as the final model-training dataset. Following sequence standardization, label harmonization, and exclusion of records lacking sufficient information for sequence-based feature computation, 28 literature-associated candidate records were retained, of which 22 sequence-defined ASOs were eligible for model development. These 22 ASOs were used exclusively for model training and internal cross-validation. The primary source associated with each dataset and the progression from initial aggregation to final model inclusion are detailed in Supplementary Table S1.

Independent experimental data were generated in this study for ASOs targeting DAZAP1 and FAM69C, two stress-granule-associated genes with emerging relevance to ALS-linked RNA-binding protein biology. These targets were not represented in the literature-derived training set and were selected to enable independent evaluation of model transfer to previously unseen gene families. Experimental measurements were produced using a consistent workflow described in Section 2.3 and were withheld from all stages of model training and feature construction. Thirty candidate ASOs targeting DAZAP1, FAM69C, and SND1 were experimentally screened. Following predefined quality-control procedures, including evaluation of technical and biological replicate consistency and exclusion of outlier Cq values, eight experimentally characterized ASOs (six targeting DAZAP1 and two targeting FAM69C) were retained for computational evaluation. These experimentally generated datasets served two purposes: (i) assessment of true out-of-distribution (OOD) generalization, and (ii) minimal family-specific calibration using a single reference ASO per new target.

To maintain interpretability and reduce confounding sources of variance, the scope of the analysis was restricted to ASOs of comparable length and design class. Sequences lacking sufficient annotation, employing substantially different chemistries, or measured under incompatible assay conditions were excluded. No data were re-weighted based on reported efficacy magnitude, and all retained measurements were treated uniformly following standardization. An overview of the literature aggregation, curated modeling datasets, independent experimental datasets, and their respective roles in model development and evaluation is provided in Table 1, with complete dataset provenance summarized in Supplementary Table S1.

**TABLE 1:** Summary of the datasets used throughout model development and evaluation. The initial literature aggregation served as the source pool for dataset curation.

Dataset category	Target genes/families	Data source	Initial records	Final observations used	Role in study
Literature aggregation	SOD1, ATXN2, C9ORF72, FUS, TGFBR2, VCP	Peer-reviewed publications and patent disclosures	150 efficacy records	Used for dataset curation	Initial literature aggregation
Literature- derived modeling dataset	SOD1, ATXN2, C9ORF72, FUS, AR, STMN2	Curated literature datasets (see Supplementary Table S1 for represented studies)	28 candidate records	22 sequence- defined ASOs	Model training and family-aware cross-validation
Independent experimental dataset	DAZAP1, FAM69C	This study	30 candidate ASOs screened	8 quality- controlled ASOs	Out-of-distribution evaluation and one-shot family calibration

Following sequence standardization and quality-control procedures, 22 literature-derived ASOs were retained for model development. Independently generated experimental ASOs were withheld from training and used exclusively for out-of-distribution evaluation and family-specific calibration. Complete dataset provenance and target-specific source references are provided in Supplementary Table S1.

### 2.3 Wet-lab ASO design and experimental measures

#### 2.3.1 Sample preparation and ASO treatment

Using 30 of the generated ASO sequences hypothesized to be the most effective, we transfected the ASO sequences into SH-SY5Y cells and extracted RNA from the transfected cells. The extracted RNA was converted to cDNA for RT-qPCR, so that we could determine which ASOs were most effective at reducing DAZAP1 and FAM69C transcription.

To prepare our ASOs, we conducted lipofection on the SH-SY5Y cells. We started with an initial seeding density of 3 × 10^5^ and observed that there was around 70% confluence of cells in the wells. We created a master mix of 100 μL of OPTI-MEM and 1 μL Lipofectamine 3,000, and added 7.5 μL of the ASO for every 300 μL of the Master Mix and let it incubate for 10 min prior to application to cells. Cells were incubated with 100 μL of the transfection mixture for 5 days prior to RNA extraction using TRIzol reagent. Once all our ASOs were prepared, we conducted RT-qPCR.

#### 2.3.2 RT-qPCR experimental design

RT-qPCR was used to quantify target gene expression. RNA samples were reverse-transcribed into cDNA, which was then amplified using gene-specific primers and a fluorescent reporter. Serial dilutions of the template were prepared to generate a standard efficiency curve, and each dilution was run in technical replicates.

After RT-qPCR, we obtained Quantification Cycle (Cq) values for each of our wells following RT-qPCR amplification (Table 2). Cq represents the cycle number at which the PCR reaction outputs a fluorescent signal, indicating the presence of the target effect.

**TABLE 2:** Efficiency calculations based on slope for DAZAP1, GAPDH, and FAM.

Primer	Slope	Efficiency
DAZ	−3.977576677	78.40595984
GAP	−4.169012789	73.72604767
FAM	−3.463829212	94.40070548

Before analysis, we performed a systematic quality control where technical replicates were evaluated for consistency. Outliers, values that substantially deviated from replicates, were identified and excluded to maintain data integrity. Standard deviations were calculated for all replicate tests with only measurements possessing acceptable low variance being retained for downstream calculations.

For primer efficiency analysis, we observed the expected inverse relationship between template concentrations and Cq values for all dilution series.

#### 2.3.3 Primer efficiency curves

We ran a primary efficiency curve to determine the amplification efficiency of each primer pair to ensure accurate quantification. Serial dilutions of cDNA were prepared for RT-qPCR using primer pairs targeting DAZAP1, GAPDH, and FAM. Each dilution was run in technical triplicates, and the Cq values were plotted against the logarithm of the initial template concentration to generate standard curves for each primer set. Primer efficiency was calculated from the slope of linear regression according to the equation:

$$Efficiency=\left(10^{-1/slope}-1\right)\times 100$$

Technical replicates were evaluated for consistency prior to any analysis. When two replicates were clustered closely and a third showed substantial deviation from the other two values, the deviating value was identified as an outlier and excluded. Removing such outliers reduced noise from technical and human errors and ensured low standard deviations, improving the reliability of the efficiency calculations. After omitting outliers, most replicates showed low standard deviations, indicating good reproducibility and precise amplification. No-template controls (NTCs) were included to assess contamination. Unexpectedly, the NTC for GAPDH did have values, suggesting that there was low-level contamination with DNA or cDNA in these wells.

The GAPDH NTC amplified at an average Cq of 26.22 (SD = 7.05, n = 2 of three replicates; the third replicate showed no amplification), compared to an average sample Cq of 15.38 (SD = 1.11) at the highest template concentration, a difference of approximately 10.8 cycles. This corresponds to more than a 1,000-fold difference in estimated starting template between the NTC and experimental samples, indicating that while trace contamination was present, it was several orders of magnitude below the true amplification signal and did not materially affect downstream quantification. NTC values were excluded from all ΔCq and ΔΔCq calculations.

In our preparation for our primer efficiency curve, we calculated log, the sample quantity, for each dilution. In qPCR, our goal was to measure the amount of DNA that increased in each PCR cycle. The relationship between amplification and cycle number follows the equation:

$$\log\left(N\right)=\log{\left(N\right)}_{0}+C\times \log\left(1+E\right)$$

The slope of this line is proportional to primer efficiency, demonstrated in Figure 3.

**FIGURE 3:**
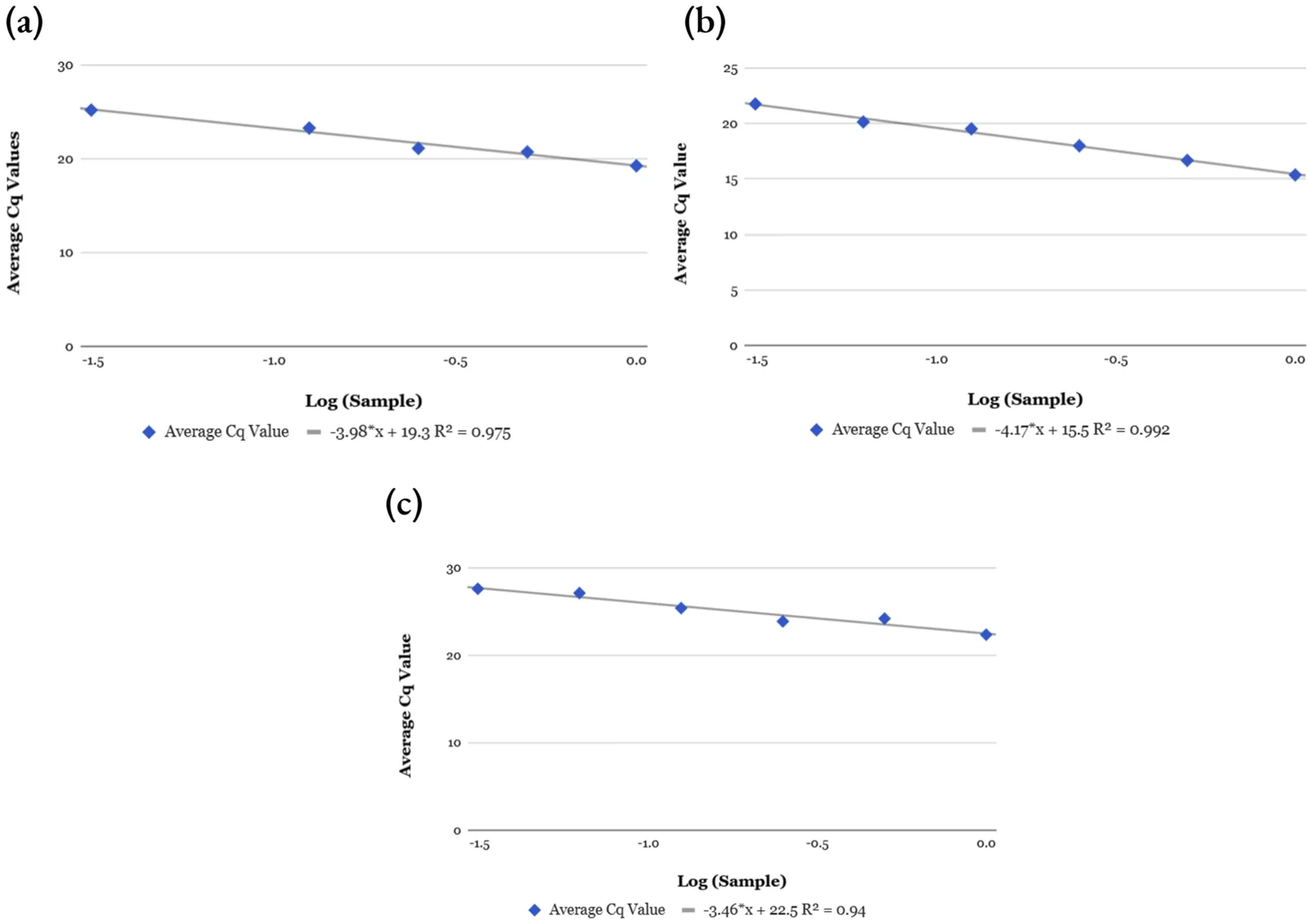
**(a)** Primer efficiency curves. Average vs. Log(sample) for DAZAP1. **(b)** Average vs. Log(sample) for GAPDH. **(c)** Average vs. Log(sample) for FAM.

For all three primers, the plot of the Cq values against the logarithm of the initial template concentration produced a linear relationship as expected for efficient primers, evident in the R2 values shown in the graphs.

Based on the slope from the primer efficiency curve, the efficiency was calculated. The efficiency of the PCR reaction indicated how well the primers amplified the target DNA in each PCR cycle. While our efficiencies were low for DAZAP1 and GAPDH, we took this limitation into account for future calculations when measuring expression fold change. The observed efficiencies for DAZAP1 (78.4%) and GAPDH (73.7%) fall below the conventionally accepted 90%–110% range for aPCR primers, which may reflect amplicon secondary structure, suboptimal primer binding kinetics, or RNA integrity. To account for suboptimal amplification, the Pfaffl method was applied to DAZAP1 quantification, which explicitly corrects for measured primer efficiency.

#### 2.3.4 Knockdown quantification

##### 2.3.4.1 DAZAP1 analysis (Pfaffl method)

For DAZAP1, we applied the Pfaffl method to calculate relative gene expression, which incorporates primer efficiency into the quantification model. This approach adjusts for non-ideal amplification and provides accurate fold-change estimates when efficiency deviates from 100%.

We calculated ΔCq values (Cq_Target_ - Cq_reference_) for the averages of each sample and control. Then, using efficiency-corrected equations, we computed relative quantities (RQ). Expression fold change was normalized to GAPDH and compared between ASO-treated samples and controls. After accounting for efficiency and removing outliers across biological replicates, there was a fold-change of approximately 78%, indicating a 22% knockdown of DAZAP1 mRNA via ASOs 20 and 23.

##### 2.3.4.2 FAM69C analysis (ΔΔCq method)

FAM69C knockdown was quantified using the ΔΔCq method. This method is appropriate when primer efficiencies approach 100% or when efficiency correction is not feasible. Raw Cq values were obtained from both FAM69C (target) and GAPDH (Reference) over treated samples, media controls, and lipofectamine controls, each with three technical and biological replicates. We calculated ΔCq values for each condition, then derived ΔΔCq by subtracting the control ΔCq from the treated ΔCq. Expression fold change was computed as 2 raised to the negative value of ΔΔCq. Because of high variability in biological replicates, only one replicate value produced consistent technical replicate values within the acceptable range. Both technical replicates from this set yielded similar Cq values. The final analysis produced expression fold changes of 50%–60% relative to controls, indicating a 40%–50% knockdown of FAM69C mRNA expression via ASOs 8 and 10.

Although the DAZAP1 and GAPDH primer efficiencies were below the ideal range, efficiency-corrected quantification (Pfaffl method) was used for DAZAP1, while only quality-controlled experimental measurements were retained for computational analysis. The experimentally generated ASOs were used exclusively for external out-of-distribution evaluation and one-shot calibration and were never incorporated into model training or hyperparameter optimization, thereby limiting the influence of experimental measurement uncertainty on model development.

#### 2.3.5 Integration into model

The experimentally measured knockdown values obtained from the RT-qPCR were integrated into the predictive framework as external reference inputs. For each ASO, the experimentally validated sequence was paired with its corresponding percent knockdown value. For the final out-of-distribution modeling and one-shot calibration pipeline, a subset of six DAZAP1 ASOs and two FAM69C ASOs were selected after passing strict wet-lab post-transfection quality controls including: minimum viability thresholds, reproducible transcript quantification across triplicates, and within-range reference gene amplification. Only wet lab measurements that met the pre-defined quality-control criteria, including consistency across technical and biological replicates and exclusion of outlier Cq values, were incorporated.

### 2.4 Label harmonization and data standardization

ASO efficacy measurements collected from published studies and collaborating laboratory experiments exhibited substantial heterogeneity in reporting format, assay context, and metadata completeness. To enable consistent modeling and cross-family evaluation, all measurements were harmonized into a unified, analysis-ready representation while preserving source origin and minimizing transformation assumptions. Protein-level measurements, when available, were used for qualitative validation but were not used as primary model labels.

Reported efficacy labels were standardized to a continuous percentage knockdown scale bounded between 0 and 100. Measurements reported as exact percentages were retained directly. When efficacy was reported as bounded ranges, the midpoint of the reported interval was used as a single representative value. Qualitative categorical labels (e.g., high, medium, low) were mapped to fixed numeric values chosen to reflect coarse ordering rather than precise quantitative equivalence. These mappings were intended solely to preserve ordinal relationships across heterogeneous literature sources rather than to imply exact quantitative equivalence. Because the independent experimental validation dataset consisted exclusively of RT-qPCR-derived quantitative knockdown measurements, the principal external performance evaluation was not dependent on these ordinal mappings. All standardized values were clipped to the biologically meaningful interval [0.100]. No labels were re-weighted or excluded based on magnitude, and no attempt was made to infer uncertainty beyond the information explicitly reported by the source. When multiple efficacy measurements were reported for the same ASO sequence (e.g., across doses, timepoints, or assay contexts), a single representative knockdown value was retained for modeling to avoid pseudo-replication and overrepresentation of individual studies. This curation reduced the effective training set size but improved statistical independence across samples.

To maintain traceability, each standardized entry retained metadata describing its origin and context, including ASO identifier, target gene, sequence, reported chemistry where available, assay modality, source domain (literature or experimental), and reference. These fields enabled stratified analysis and family-aware evaluation without conflating measurements across incompatible contexts. A unified schema was applied to both literature-derived and experimentally obtained data to ensure identical downstream treatment.

Because ASO efficacy datasets are typically small and family-structured, no imputation was performed for missing metadata fields beyond sequence and target identity, which were required for inclusion. Measurements lacking sufficient annotation to support sequence-based feature computation or family assignment were excluded prior to modeling. Importantly, all harmonization steps were performed before any model training, feature selection, or evaluation, and standardized labels were fixed for all subsequent analyses.

The resulting standardized dataset served as the sole source of training, calibration, and evaluation labels for the predictive framework. Detailed mapping rules, example transformations from raw reported labels to standardized values, and per-family summary statistics are provided in the Supplementary Methods S3 and Supplementary Table S4.

### 2.5 Feature engineering and biophysical representations

ASO sequences were transformed into a compact set of biologically motivated features designed to capture determinants of RNase H–dependent knockdown efficacy while remaining interpretable under small-data constraints. Feature design was guided by established principles of ASO activity, including sequence composition, gapmer architecture, RNA-DNA hybridization thermodynamics, and target RNA accessibility (Shen and Corey, 2018; Crooke et al., 2021; Hörberg et al., 2024).

Sequence-level compositional features were computed directly from the ASO nucleotide sequence and included length, overall GC content, CpG density, and maximal homopolymer run length. To reflect gapmer-specific design considerations, regional GC content was computed separately for the 5′ wing, central DNA gap, and 3′ wing, consistent with the canonical 5-10-5 architecture commonly used in RNase H–active ASOs. In addition, low-order di- and tri-nucleotide motif frequencies were included to capture local sequence patterns previously associated with antisense activity and off-target effects.

Although information on ASO chemistry and modification patterns was retained during dataset curation when available, individual chemical modifications were not encoded as independent numerical features in the gradient boosting model. Instead, the analysis was restricted to comparable RNase H-active gapmer ASOs, allowing sequence-derived descriptors, target-context features, thermodynamic descriptors, RNA accessibility measures, RNase H-related heuristic features, and principal components of k-mer composition to capture the principal determinants of knockdown efficacy while minimizing chemistry-driven confounding. The resulting numerical feature vectors supplied to the gradient boosting model consisted exclusively of sequence-derived and biophysically derived descriptors. Experimental metadata, target-family labels, assay information, and chemical modification annotations were retained only for dataset curation and downstream evaluation and were not used as predictive model inputs.

To incorporate biophysical constraints on productive ASO binding, thermodynamic estimates of duplex stability were computed using nearest-neighbor thermodynamic calculations implemented through ViennaRNA RNAduplex, which estimates the free energy of duplex formation between complementary sequences (Lorenz et al., 2011). These estimates were used as computational descriptors of relative ASO-target binding propensity. However, chemically favorable hybridization alone does not guarantee functional activity if the target site is structurally inaccessible. RNA secondary-structure accessibility was therefore estimated using RNAplfold, an ensemble-based partition-function folding algorithm from the ViennaRNA package that computes nucleotide unpaired probabilities and local accessibility across the target transcript (Bernhart et al., 2006; Lorenz et al., 2011).

Specifically, duplex stability was quantified using nearest-neighbor free-energy calculations (ΔG_duplex_), while RNA accessibility was represented by the energetic cost of opening the target site (ED) derived from RNAplfold. These complementary biophysical descriptors formed the basis of the effective binding representation used during model development. Complete mathematical definitions, including the formulation of ΔG_eff_, parameter settings, and implementation details, are provided in Supplementary Methods S4.

Hybridization and accessibility terms were combined into a single effective binding proxy, defined conceptually as the difference between duplex formation free energy and the energetic penalty associated with RNA structural opening. This formulation reflects the requirement that RNase H–active ASOs must both hybridize stably and access their target site to form a productive RNA–DNA heteroduplex. Where RNA accessibility estimates were unavailable due to sequence length or structural constraints, models were trained using sequence-only and thermodynamic features to ensure consistent treatment across datasets.

All features were calculated using fixed parameter settings and standardized computational tools prior to model training. Feature computation was performed once per dataset version and cached as a versioned artifact to ensure that all model variants and evaluations operated on identical inputs. This approach prevented inadvertent information leakage and enabled reproducible comparison across modeling choices. Formal mathematical definitions, software implementation details, parameter settings, and feature derivations are provided in Supplementary Methods S4 and Supplementary Tables S5, S6.

### 2.6 Predictive modeling and family-aware calibration

Predictive modeling was performed using a regression framework designed to operate reliably under small-sample, family-structured conditions typical of ASO efficacy datasets. Because the available data comprise heterogeneous target gene families with limited per-family sample sizes, we prioritized model classes that capture low-order non-linear interactions while limiting overfitting and preserving interpretability.

We employed a gradient-boosting regression model with shallow decision trees as base learners. Gradient boosting incrementally fits weak learners to residual errors, enabling the model to represent interactions among sequence-derived and biophysical features (e.g., motif composition × regional GC content or hybridization energetics × accessibility) without requiring high-capacity architectures. Tree depth, learning rate, and ensemble size were constrained to reduce variance and ensure stable behavior across small folds. Alternative regression approaches, including linear models and kernel-based methods, were explored during preliminary analyses but were not selected due to reduced flexibility or sensitivity to feature scaling in this setting (Smola and Schölkopf, 2004).

Because ASO efficacy measurements are strongly structured by target gene family, systematic shifts in baseline knockdown levels were observed across families even after feature standardization. To account for this effect during model transfer to new targets, we incorporated a family-aware calibration step. For a previously unseen target family, the calibration procedure applies an additive offset derived from a single reference ASO measured experimentally within that family. This offset corrects systematic bias in predicted knockdown magnitude while preserving the relative ranking of ASOs within the family.

Formally, let ŷᵢ denote the model-predicted knockdown efficacy for ASO i, and let y_c_ and ŷ_c_ denote the observed and predicted knockdown values, respectively, for a designated calibrator ASO within the same target family. The calibrated prediction is defined:

$${ŷ}_{i_{\mathrm{cal}}}={ŷ}_{i}+\left(y_{c}-{ŷ}_{c}\right)$$

This additive calibration approach was selected over monotonic re-mapping methods, such as isotonic regression, which can become unstable or degenerate when applied to very small family sizes. Calibration was applied only during evaluation on held-out families and was never used during model training.

All models were trained using fixed feature representations generated prior to model fitting and cached as versioned artifacts. Hyperparameters were selected using internal cross-validation on training families only, and no information from held-out or out-of-distribution families was used during model selection or calibration parameterization. Random seeds were fixed to ensure reproducibility across runs.

### 2.7 Evaluation strategy and performance metrics

Model performance was evaluated using a validation strategy designed to reflect the family-structured and small-sample nature of ASO efficacy datasets. All evaluation procedures were defined prior to analysis and enforced strict separation between training, calibration, and test data to prevent information leakage.

Internal model assessment was performed using family-aware cross-validation. When sufficient data were available, stratified k-fold cross-validation was applied such that ASOs targeting the same gene family were confined to a single fold. For families with very small sample sizes, leave-one-out cross-validation (LOOCV) was used within the training set. Hyperparameter tuning and model selection were conducted exclusively within training folds and did not involve held-out families.

To assess generalization beyond literature-derived targets, true OOD evaluation was performed by withholding entire target gene families from model training. In these experiments, all ASOs associated with a given target gene (e.g., DAZAP1 or FAM69C) were excluded from training and feature selection. The trained model was then applied to the held-out family, optionally followed by family-specific calibration using a single reference ASO as described in Section 2.6. No information from OOD families was used during model fitting or hyperparameter selection.

Model accuracy was quantified primarily using the mean absolute error (MAE), defined as the mean of the absolute differences between predicted and observed knockdown efficacy values. MAE was selected as the primary metric because it provides an interpretable measure of average prediction error in percentage knockdown units and is less sensitive to outliers than squared-error–based metrics (Willmott and Matsuura, 2005). For completeness, the predictive coefficient of determination (R^2^) was calculated directly from the observed and predicted knockdown values as

$$R^{2}=1-\frac{\Sigma_{i}{\left(y_{i}-\hat{y_{i}}\right)}^{2}}{\Sigma_{i}{\left(y_{i}-{\bar{y}}_{i}\right)}^{2}}$$

where y_i_ and y^^^_i_ denote the observed and predicted knockdown values, respectively, and yˉ denotes the mean observed knockdown. This metric was computed without fitting a separate regression line; therefore, deviations between predicted and observed values directly reduced the reported score. Spearman’s rank correlation coefficient (ρ) was used separately to assess monotonic agreement between predicted and observed ASO rankings.

Uncertainty in performance estimates was quantified using nonparametric bootstrap resampling. For each evaluation setting, ASOs were resampled with replacement, and performance metrics were recomputed across bootstrap replicates to obtain bootstrap-derived 95% confidence intervals. Bootstrap resampling was applied separately to overall performance estimates and to per-family out-of-distribution results, providing an empirical characterization of uncertainty appropriate for small-sample biological datasets.

All evaluations were conducted using fixed data partitions and versioned feature artifacts generated prior to model training. Random seeds were fixed for cross-validation splits and bootstrap resampling to ensure reproducibility. Performance comparisons across modeling variants were made using identical training and test splits.

#### 2.7.1 Validation of one-shot calibration via leave-one-family-out simulation on literature families

To assess the behavior and reliability of one-shot family calibration independently of the limited experimental validation set, we performed a repeat-based leave-one-family-out (LOFO) simulation using literature-derived ASO efficacy data. In this analysis, all ASOs targeting a given gene family were held out as an out-of-distribution test set, while the predictive model was trained on ASOs targeting all remaining families.

Uncalibrated knockdown predictions were first generated for the held-out family. A single ASO from the held-out family was then randomly selected as a calibration reference, and an additive offset was computed as the difference between the observed and predicted knockdown for this reference ASO. This offset was applied uniformly to the remaining ASOs within the held-out family, yielding calibrated predictions without retraining or modification of model parameters. Calibration performance was evaluated by comparing MAE before and after calibration on the remaining ASOs.

To characterize variability arising from calibrator choice under small-sample conditions, this procedure was repeated 1,000 times per family, each time selecting a different calibrator ASO at random. For each family, we report the distribution of calibration-induced MAE changes, including the mean and median improvement, the fraction of repeats in which calibration reduced error, and repeat-based empirical 95% confidence intervals derived from the observed improvement distribution. Families with fewer than two ASOs were excluded from this analysis.

This simulation provides a controlled validation of the one-shot calibration strategy under realistic small-n conditions and complements the experimental evaluation by isolating the calibration mechanism from wet-lab sampling limitations.

## 3 Results

### 3.1 Dataset composition and scope of evaluation

To ensure statistical independence and avoid overrepresentation of individual studies, a single standardized %KD was retained per ASO sequence following the label harmonization procedures described in Methods Section 2.4 and Supplementary Methods S3.

The final modeling dataset comprised literature-derived ASOs targeting ALS-relevant genes, including canonical disease targets such as SOD1, ATXN2, and C9ORF72, as well as downstream or pathway-associated genes retained under their reported symbols (e.g., AR, STMN2). These literature-derived ASOs were used exclusively for model training and internal validation. Similarly, experimentally measured ASOs targeting DAZAP1 and FAM69C were generated using a consistent RT-qPCR workflow and were withheld entirely from model training and hyperparameter selection. These experimental targets therefore served as true OOD families for evaluating cross-family transfer and calibration behavior.

Per-target sample sizes varied substantially, reflecting both ASO literature and practical constraints of experimental validation. This heterogeneity motivated the family-aware evaluation strategies and uncertainty quantification used throughout. Dataset composition by target family, including raw aggregation counts, curated modeling counts, source domains, and primary knockdown assay modality, is summarized in Supplementary Table S1.

### 3.2 Mechanistic modeling components reduce external wet-lab prediction error

We next evaluated whether incorporating biophysically motivated modeling components improves prediction of ASO knockdown efficacy when assessed on independent experimental data. As previously detailed, we compared a set of representative models that incorporate increasing amounts of mechanistic information and evaluated all models under identical conditions on a fixed external benchmark consisting of eight independently generated wet-lab ASOs.

Model configurations were constructed to incorporate increasing levels of biological information, beginning with sequence-derived features and progressively adding RNA-DNA hybridization thermodynamics and RNA accessibility. For each configuration, a single representative model was evaluated on the same set of experimental ASOs. Prediction accuracy was quantified using MAE, defined as the average absolute difference between predicted and observed transcript-level %KD across the eight ASOs.

As shown in Figure 4, models trained using sequence-derived features alone exhibited relatively high external prediction error, indicating limited generalization from literature-derived data to experimental targets. Incorporating RNA-DNA hybridization thermodynamics resulted in a substantial reduction in MAE, consistent with the expectation that duplex stability is a first-order determinant of RNase H-mediated knockdown. This step resulted in the largest single improvement in external prediction accuracy among the evaluated configurations.

**FIGURE 4:**
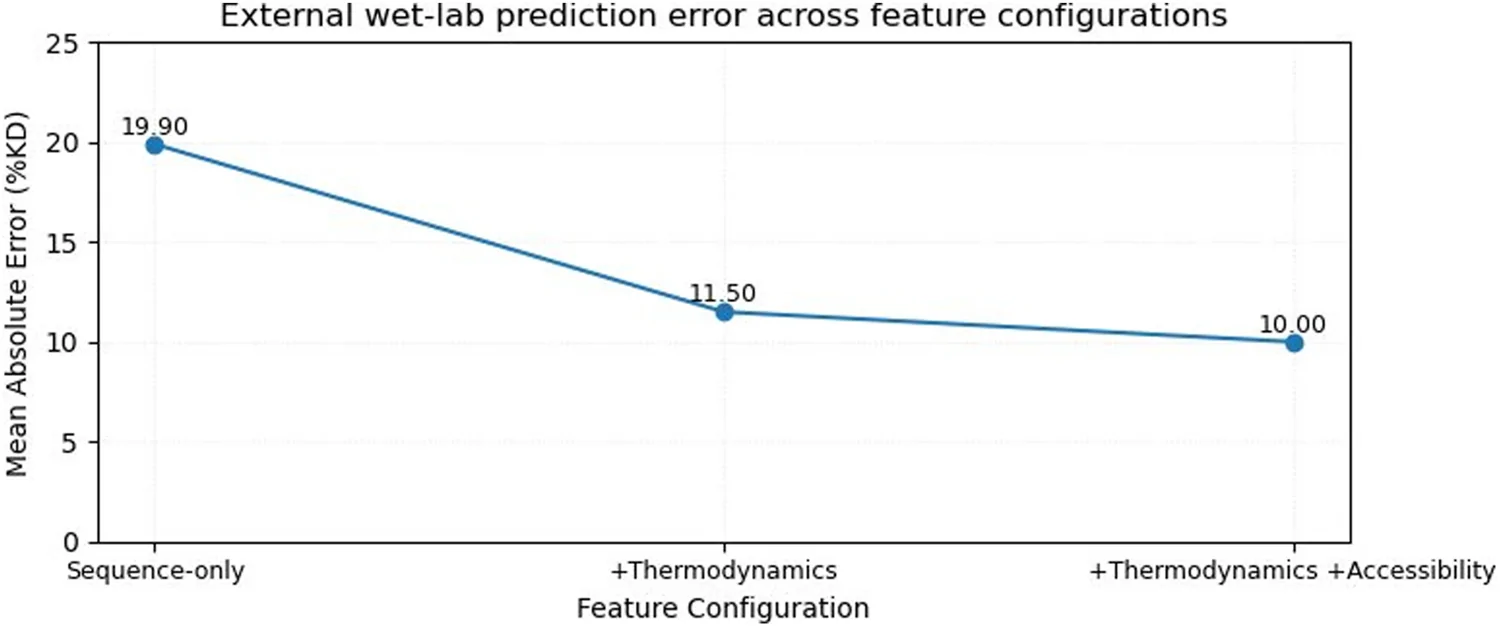
MAE for ASO knockdown predictions on an independent wet-lab dataset using progressively more informative feature sets. Adding thermodynamic features produced the largest reduction in mean absolute error, while incorporation of RNA accessibility provided a further improvement in external prediction accuracy. Uncertainty associated with the external benchmark is quantified separately via bootstrap resampling (see Supplementary Methods S6).

Importantly, all MAE values reported in this comparison were computed on the same fixed experimental ASOs using an identical error definition, ensuring that differences reflect modeling choices rather than evaluation variability. Uncertainty associated with the external benchmark was estimated separately using bootstrap resampling of the experimental ASOs (Supplementary Figure S7C and Supplementary Results S7).

Together, these results demonstrate that incorporating mechanistic determinants of ASO activity, particularly hybridization thermodynamics and target accessibility, improves generalization to experimental data. Sequence-derived features provide a useful baseline, but biophysically informed representations yield lower external prediction error and more accurate modeling of RNase H-active knockdown behavior.

### 3.3 Uncalibrated models exhibit family-specific prediction bias

Although mechanistic feature augmentation substantially reduced external prediction error, residual bias remained when predictions were examined at the level of individual target families. To assess whether these errors reflected random noise or systematic effects, we analyzed predicted versus observed knockdown efficacy for the experimental ASOs prior to any family-specific calibration.

As shown in Figure 5, uncalibrated predictions exhibited clear family-dependent offsets. In particular, predictions for both DAZAP1 and FAM69C clustered within a relatively narrow band of predicted knockdown values, despite spanning a broader range of observed transcript-level knockdown. This behavior indicates that, while the model preserved relative ordering to some extent, absolute prediction scale differed systematically across target families.

**FIGURE 5:**
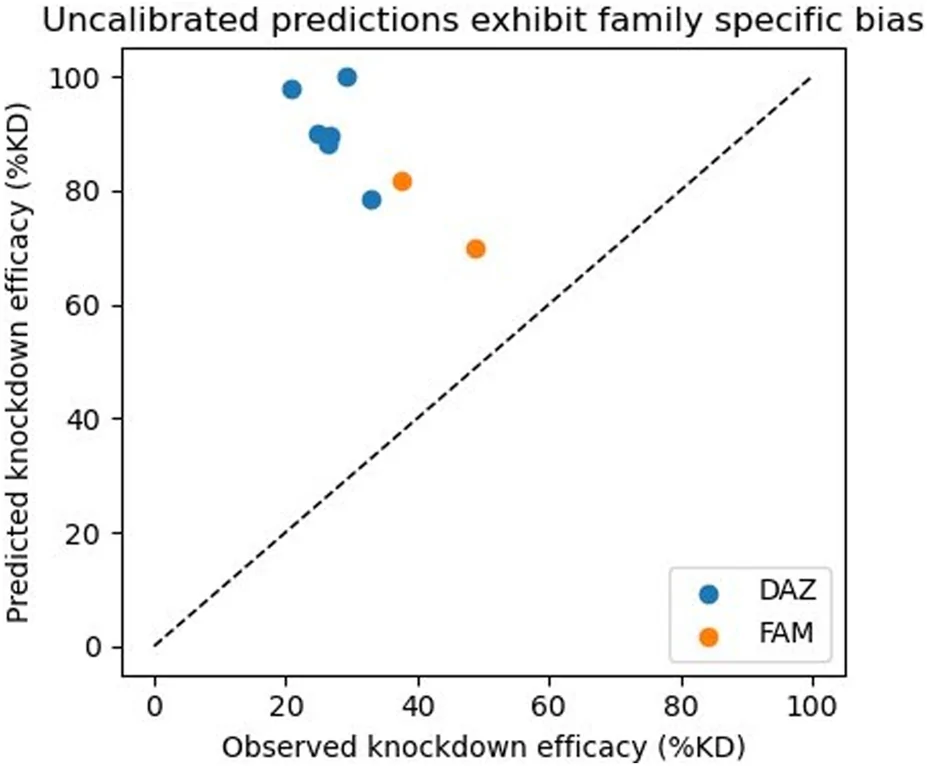
Predicted versus observed transcript-level %KD for independent DAZAP1 and FAM69C ASOs before family-specific calibration. Predictions show systematic offsets from the identity line, indicating target-family-specific bias despite preserving general trends in knockdown efficacy. The complete observed and predicted values for each experimental ASO are provided in Supplementary Table S3.

Visualization of uncalibrated predictions highlights this effect, with predicted values deviating consistently above or below the identity line for individual families (Figure 5). Residuals computed as the difference between predicted and observed knockdown further confirm the presence of structured, family-level error rather than purely stochastic variation.

### 3.4 One-shot family calibration corrects systematic bias while preserving rank

Calibration was implemented as a *post hoc* additive offset applied to model predictions, computed as the difference between the observed and predicted knockdown efficacy for the designated calibrator ASO. This adjustment corrects family-level intercept shifts without modifying the learned model parameters or altering relative ordering among ASOs within the family. Importantly, calibration was applied only during evaluation and did not involve retraining, feature reweighting, or additional data beyond the single reference measurement.

Applying this one-shot calibration substantially improved alignment between predicted and observed knockdown efficacy for both experimental target families. As shown in Figure 6, calibrated predictions closely follow the identity line, in contrast to the pronounced offsets observed prior to calibration. Correspondingly, mean absolute error on the external wet-lab benchmark decreased dramatically following calibration, reflecting a correction of systematic scale bias rather than incremental refinement of individual predictions.

**FIGURE 6:**
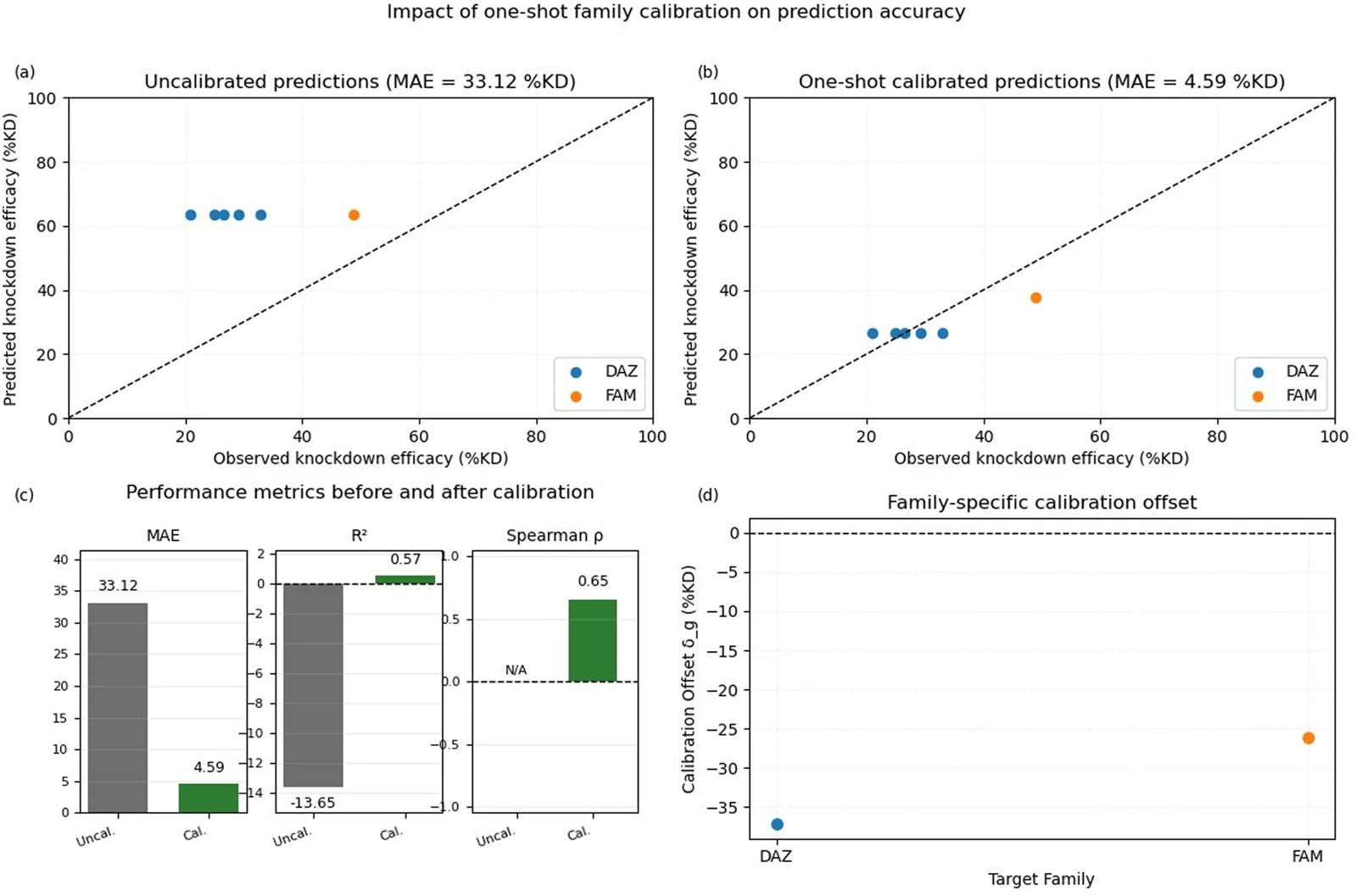
Impact of one-shot family calibration on external prediction performance. **(a)** Uncalibrated predictions for the non-calibrator evaluation ASOs. **(b)** Predictions following one-shot family calibration. **(c)** Comparison of MAE, R^2^, and ρ before and after calibration. Spearman’s rank correlation was not applicable for the uncalibrated predictions because the predicted values were effectively constant across the evaluation ASOs. **(d)** Family-specific additive calibration offsets applied during one-shot calibration. The quantitative values underlying all experimental predictions are provided in Supplementary Table S3.

Crucially, this improvement in absolute accuracy was achieved without degrading rank-based performance. Spearman’s rank correlation remained stable before and after calibration, indicating that the model’s ability to prioritize ASOs within a target family was preserved. This behavior is consistent with the design of the additive calibration approach, which adjusts global scale while leaving relative differences intact.

Inspection of the learned calibration offsets further supports this interpretation. Estimated offsets differed across target families, consistent with family-specific baseline shifts in knockdown efficacy, but were applied uniformly across ASOs within each family. These offsets provide a compact summary of family-level adjustment and can be interpreted as correcting systematic differences in transcript context or assay response not fully captured by sequence-derived features alone.

Evidently, a single reference ASO per target family is sufficient to correct systematic prediction bias when transferring models to new experimental contexts. This one-shot calibration strategy offers a practical compromise between zero-shot generalization and full retraining, enabling accurate absolute knockdown prediction while maintaining model simplicity and interpretability.

#### 3.4.1 Validation of one-shot calibration via LOFO simulation on literature families

While one-shot family calibration substantially improves alignment between predicted and observed knockdown efficacy in experimental evaluations, validation based on a small number of wet-lab ASOs is inherently limited. To assess the robustness and expected behavior of this calibration strategy independently of experimental sample size, we performed a repeat-based LOFO simulation using literature-derived ASO families.

LOFO simulations reveal strongly family-dependent calibration behavior (see Supplementary Table S9). For some families, calibration consistently reduces absolute prediction error, indicating the presence of a systematic family-level scale bias that can be corrected using a single reference measurement. In contrast, other families exhibit mixed or negative effects, reflecting substantial within-family heterogeneity that is not well captured by a uniform offset. Importantly, calibration does not uniformly improve performance across families, demonstrating that the approach behaves conservatively and does not artificially inflate accuracy when its assumptions are not met.

These simulations provide an independent validation of the one-shot calibration strategy under realistic small-n conditions and contextualize experimental results by distinguishing systematic family-level bias from intrinsic biological variability.

### 3.5 Out-of-distribution performance and uncertainty across experimental target families

We next evaluated the performance of the calibrated model on independently generated experimental ASOs to assess OOD generalization and quantify uncertainty under small family sizes. These analyses were conducted exclusively on wet-lab measurements for DAZAP1 and FAM69C, which were not used for model training or hyperparameter selection and therefore represent true transfer to previously unseen target families.

After one-shot family calibration, prediction accuracy improved substantially for both experimental targets (Figure 7). However, the magnitude and stability of performance differed across families, reflecting differences in sample size and inherent variability. For DAZAP1 (n = 6), calibrated predictions exhibited low absolute error with relatively narrow uncertainty intervals, indicating stable generalization under limited but nontrivial family size. In contrast, performance estimates for FAM69C (n = 2) showed greater variability, consistent with the statistical instability expected when evaluating rank- and variance-based metrics on extremely small sample sets.

**FIGURE 7:**
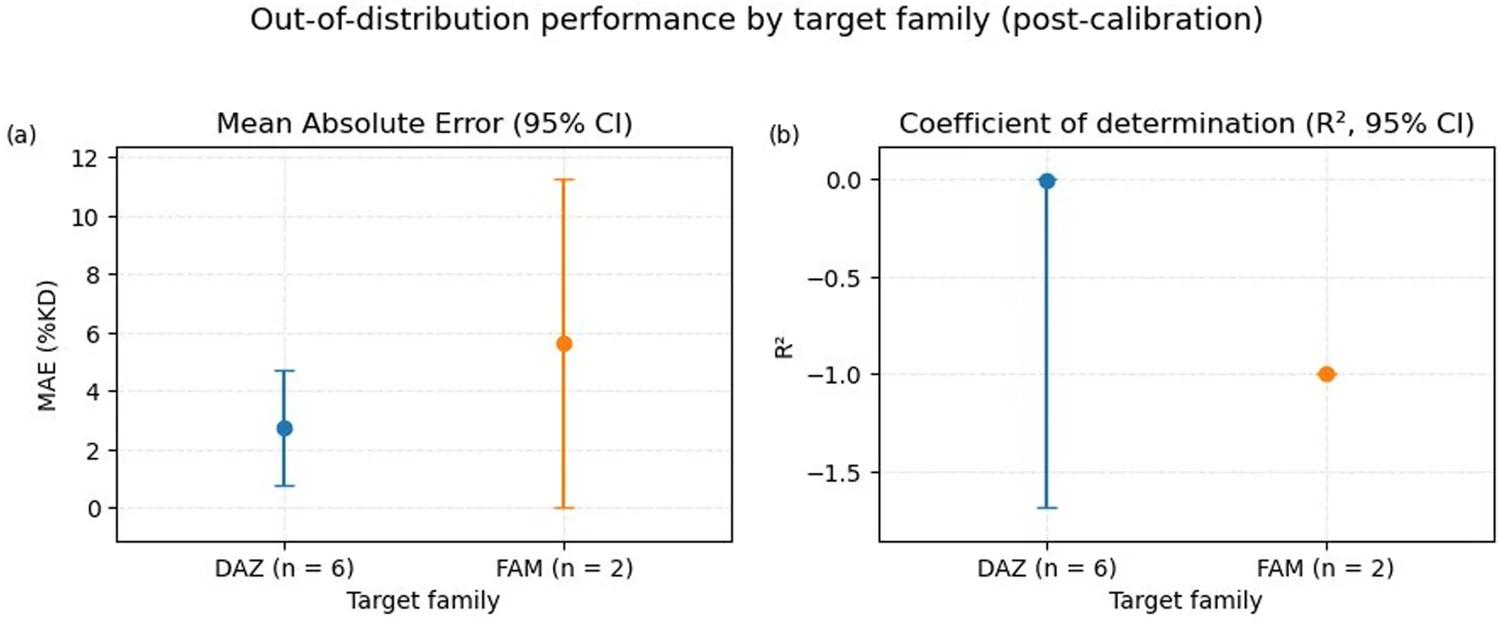
Post-calibration out-of-distribution performance by experimental target family. **(a)** MAE for each experimental target family with bootstrap-derived 95% confidence intervals. **(b)** R^2^ with bootstrap-derived 95% confidence intervals. Spearman’s rank correlation was not reported on a per-family basis because stable estimation was not possible under the available family-level sample sizes.

To characterize uncertainty more explicitly, we examined residual distributions and bootstrap-based confidence intervals for each family (Supplementary Figure S7 and Supplementary Results S7). Residuals for DAZAP1 were centered near zero following calibration, with no clear systematic trend across the observed knockdown range. In contrast, residuals for FAM69C were more dispersed, and bootstrap distributions exhibited broader tails, reflecting the sensitivity of MAE and correlation estimates at very small n. Importantly, this variability reflects uncertainty in evaluation rather than evidence of model failure, as the same calibration procedure was applied uniformly across families.

Rank-based performance metrics were interpreted conservatively in this setting. While Spearman’s correlation provided a useful qualitative check on rank preservation, its numerical value was unstable for FAM69C due to the limited number of data points and was therefore not emphasized as a primary indicator of performance. MAE remained the most interpretable and robust metric for assessing absolute prediction accuracy under these conditions.

### 3.6 Model interpretability and biological consistency

To assess whether predictive performance was driven by biologically meaningful signals rather than spurious correlations, we examined model interpretability using permutation-based feature importance and partial dependence analyses. These analyses were performed on the final calibrated gradient boosting model and are intended to characterize global feature reliance rather than establish causal relationships.

As shown in Figure 8, permutation importance analysis revealed that a small number of global sequence composition features accounted for the majority of predictive signals. In particular, principal components derived from 5-mer and 6-mer sequence composition exhibited the largest increases in MAE when permuted, indicating strong functional dependence of the model on holistic sequence architecture. These components capture orthogonal axes of nucleotide composition and motif enrichment across the full ASO sequence. As also illustrated in Figure 8, thermodynamic descriptors, seed-region statistics, and local GC-content features exhibited comparatively lower marginal importance. This pattern does not imply that these features are biologically irrelevant; rather, their information content appears to be partially redundant with global k-mer composition once correlated descriptors are compressed into orthogonal components.

**FIGURE 8:**
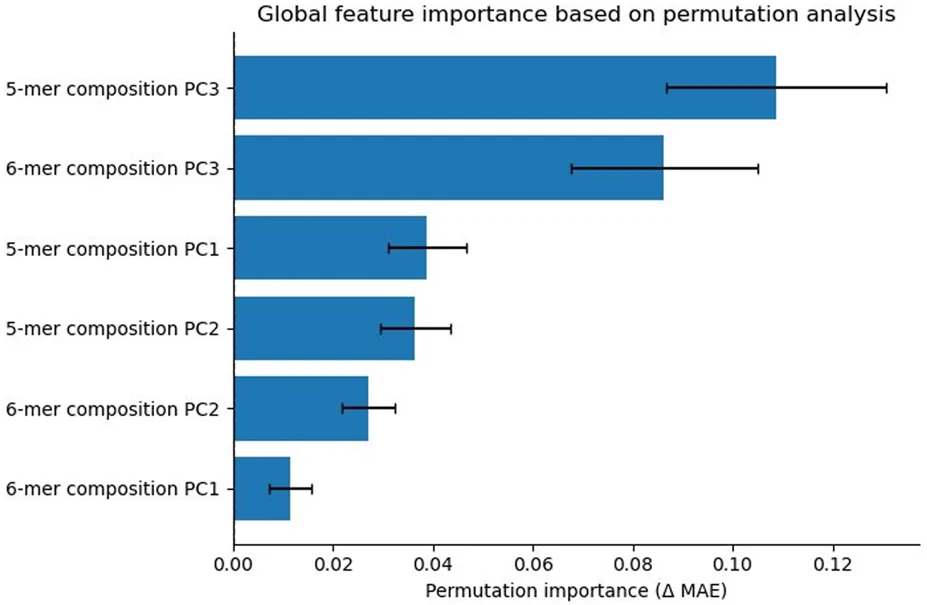
Global feature importance based on permutation analysis. Permutation importance of PCA-compressed sequence and biophysical features in the final gradient boosting model, measured as the increase in MAE following feature permutation. Importance was computed on the training distribution to assess global model reliance. Principal components of k-mer composition dominate predictive signal, whereas thermodynamic, seed-region, and local GC features exhibit lower marginal importance, reflecting redundancy with global sequence architecture. Error bars denote variability across repeated permutations. Only features with non-zero permutation importance are shown.

Partial dependence plots for the most influential numeric features further illustrated biologically plausible response patterns. Predicted knockdown efficacy increased monotonically with more favorable sequence composition and binding-related feature values, while accessibility-informed features exhibited threshold-like behavior consistent with the requirement that target sites exceed minimal accessibility for productive RNase H-mediated binding. These relationships were smooth and stable, with no abrupt discontinuities suggestive of overfitting to individual ASOs.

Importantly, interpretability analyses were qualitatively unchanged following one-shot family calibration. Calibration corrected family-level scale mismatch without altering the learned feature–response relationships, indicating that improved absolute accuracy arose from intercept adjustment rather than modification of underlying sequence-biophysical dependencies.

## 4 Discussion

The results presented in this study demonstrate that ASO knockdown efficacy can be predicted with useful accuracy under realistic small-data conditions by combining biologically grounded feature representations with a minimal, family-aware calibration strategy. Rather than relying on high-capacity models or large homogeneous datasets, this framework explicitly addresses the constraints that characterize ASO discovery in practice: limited measurements per target gene, heterogeneous literature sources, and systematic shifts across gene families and experimental contexts.

A central finding is that mechanistic feature augmentation materially improves generalization to independent experimental data. Models based solely on sequence composition capture coarse trends in ASO activity but exhibit substantial external error. Incorporating RNA-DNA hybridization thermodynamics yields a large reduction in prediction error, consistent with the established role of duplex stability in RNase H-mediated knockdown. Further improvements arise when target RNA accessibility is included, reflecting the requirement that favorable binding energetics alone are insufficient if the target site is structurally occluded. Together, these results support the view that effective ASO activity depends on an interplay between sequence architecture, binding thermodynamics, and structural context, and that explicitly encoding these factors improves predictive performance beyond sequence heuristics.

Despite these gains, uncalibrated models exhibited systematic target-family–specific bias when applied to previously unseen genes. Importantly, this bias manifested primarily as a scale mismatch rather than a loss of relative ordering, indicating that the model learned transferable sequence–biophysical relationships but lacked information about family-specific baselines. The one-shot calibration strategy introduced here directly addresses this failure mode. By using a single experimentally measured reference ASO per target family to apply a *post hoc* additive offset, calibration corrected the absolute prediction scale while preserving rank-based prioritization. This approach differs from retraining or multi-shot adaptation strategies and is deliberately minimal, reflecting a realistic experimental workflow in which only one or a small number of wet-lab measurements are available for a new gene. LOFO simulations on literature-derived ASO families further indicate that one-shot calibration is not universally beneficial, but instead provides consistent gains primarily when prediction error is dominated by a family-level intercept shift. In families characterized by substantial within-family heterogeneity, calibration may be neutral or detrimental, a behavior that is expected biologically and supports selective rather than indiscriminate application of calibration.

OOD evaluation on experimentally measured ASOs highlights both the promise and the limitations of this approach. For DAZAP1, which had a modest number of experimental ASOs, calibrated predictions were stable and exhibited low absolute error. In contrast, performance estimates for FAM69C showed greater uncertainty, consistent with the inherent instability of evaluation metrics at extremely small sample sizes. These observations underscore the importance of uncertainty quantification when interpreting model outputs in sparse biological regimes. MAE was therefore used as the primary performance metric due to its robustness and interpretability in small-sample settings, whereas rank-based metrics were interpreted conservatively given known instability under limited data.

Interpretability analyses further support the biological plausibility of the learned models. Permutation-based feature importance revealed that predictive signal is dominated by global sequence architecture, as summarized by principal components of k-mer composition, with thermodynamic and accessibility-related features contributing correlated, secondary information. This finding does not diminish the biological relevance of thermodynamic or structural descriptors; rather, it suggests that, once correlated sequence patterns are compressed into orthogonal representations, much of the relevant biophysical information is already embedded in holistic sequence composition. Partial dependence analyses showed smooth, monotonic or threshold-like relationships between key features and predicted knockdown efficacy, consistent with established principles of ASO activity and providing reassurance that improved performance does not arise from spurious correlations.

### 4.1 Study limitations

Several limitations of this study should be acknowledged. First, the overall dataset remains small, and performance estimates for out-of-distribution families are sensitive to sample size. While this reflects the realities of ASO research, additional experimental measurements per target would improve both model calibration and uncertainty estimation. In addition, standardization of qualitative efficacy descriptors into ordinal numerical values may introduce uncertainty because such mappings cannot fully capture differences in reporting schemes across heterogeneous literature sources. This limitation primarily affects the literature-derived training dataset; however, the principal external validation was performed using independently generated quantitative RT-qPCR measurements, reducing its influence on the primary conclusions of this study. Second, knockdown efficacy was defined at the transcript level, which provides a mechanistically upstream readout of RNase H activity but does not directly capture protein-level dynamics. Protein turnover rates, translational regulation, and temporal effects may modulate downstream outcomes, and future work incorporating matched protein measurements or longitudinal data would enable more comprehensive evaluation. Third, although the feature set was deliberately compact and interpretable, it does not capture all factors known to influence ASO performance, such as chemical modifications, delivery efficiency, or cellular uptake, which were intentionally controlled or excluded to reduce confounding. One-shot calibration is not universally beneficial across all target families; in some cases it yields neutral or negative improvement, indicating that a single offset cannot capture heterogeneous within-family effects. In addition, sub-optimal primer efficiency for DAZAP1 and GAPDH introduces additional uncertainty into Cq-to-fold-change conversion despite efficiency correction, and future work should prioritize redesigning these primer pairs to fall within the standard 90%–110% range.

Despite these study limitations, the framework presented here offers a practical pathway for integrating computational modeling with experimental ASO design. By leveraging heterogeneous literature data, encoding biologically meaningful features, and applying minimal calibration informed by a single experimentally measured reference ASO per target family, the model can be transferred to new targets in a manner that is both interpretable and experimentally feasible. This approach complements, rather than replaces, wet-lab screening and provides a principled means to prioritize candidates when exhaustive experimental testing is impractical.

## 5 Conclusion

This study presents a practical and biologically grounded framework for predicting ASO knockdown efficacy under realistic small-data conditions. By integrating compact sequence-based representations with thermodynamic and accessibility-informed features, the model captures key determinants of RNase H-active ASO activity while remaining interpretable and robust in the presence of heterogeneous data sources.

A central contribution of this work is the demonstration that systematic target-family-specific bias, rather than a failure of learned sequence-biophysical relationships, limits zero-shot generalization to new genes. A minimal one-shot calibration strategy, using a single experimentally measured reference ASO per target family, can effectively correct this bias, substantially improving absolute prediction accuracy without retraining or loss of within-family ranking. This approach reflects a realistic experimental workflow and provides a practical bridge between literature-trained models and new experimental targets.

OOD evaluation on independently generated wet-lab ASOs highlights both the promise and the limitations of predictive modeling in sparse biological regimes. While performance is stable when modest experimental data are available, uncertainty increases sharply at very small sample sizes, underscoring the importance of cautious interpretation and explicit uncertainty quantification.

Together, these findings illustrate how mechanistic insight, conservative modeling choices, and minimal experimental anchoring can extend the utility of computational approaches in ASO design. More broadly, this framework provides a template for sequence-based therapeutic modeling in data-limited settings, complementing experimental screening and supporting informed candidate prioritization.

## Data Availability

The datasets presented in this study can be found in online repositories. The names of the repository/repositories and accession number(s) can be found in the article/supplementary material and coalesced in the GitHub repository here: https://github.com/rguha-cs/small-data-aso-ml-pipeline.
