# Supplementary material for: Biophysics-informed computational framework for predicting antisense oligonucleotide knockdown efficacy under small-data constraints
Source: Front Syst Biol. 2026 Oct 14;6:1921636. doi: 10.3389/fsysb.2026.1921636 (PMC13713387; doi:10.3389/fsysb.2026.1921636)
Supplement: Supplementary file 1 [file DataSheet1.pdf]

Data and analysis code are available at: https://github.com/rguha-cs/small-data-aso-ml-pipeline

---

Supplementary Methods S1. Dataset construction and preprocessing

S1.1 Literature data collection

Literature-derived antisense oligonucleotide (ASO) efficacy measurements were collected from peer-reviewed publications reporting RNase H-active ASOs targeting ALS-associated or neurodegeneration-related genes. Eligible studies reported sufficient sequence information for feature computation together with transcript-level knockdown measurements or equivalent molecular efficacy endpoints.

Studies were included if they:

- reported ASO sequence information suitable for computational feature extraction;
- targeted a defined transcript or gene associated with neurodegeneration or RNA-binding protein biology;
- quantified knockdown using transcript-level assays (e.g., RT-qPCR or branched-DNA assays); and
- investigated RNase H-active ASO architectures.

Studies were excluded if sequence information was incomplete, ASOs employed fundamentally different mechanisms (e.g., steric-blocking ASOs or siRNA), or efficacy was reported only through downstream functional phenotypes without molecular knockdown measurements.

Protein-level measurements reported in some studies were retained for qualitative interpretation but were not used as quantitative model labels. The initial literature aggregation comprised 150 efficacy records extracted from peer-reviewed publications and patent disclosures spanning multiple target genes, assay systems, and reporting formats. This aggregation served as the source pool for subsequent curation rather than as the final modeling dataset. The literature sources represented within each modeled target dataset are summarized in Supplementary Table S1.

S1.2 Dataset curation

Initial literature aggregation contained multiple measurements for many ASO sequences arising from different doses, tissues, assay conditions, or experimental timepoints.

To avoid pseudo-replication during model development, one representative knockdown measurement was retained for each unique ASO sequence.

Entries lacking sufficient sequence annotation or target assignment for feature computation were excluded before modeling.

Following sequence standardization and quality-control filtering, 28 literature-associated candidate records were retained. Six records lacked publicly disclosed ASO sequences or sufficient sequence information for feature computation and were therefore excluded from sequence-based modeling. The final literature-derived modeling dataset comprised 22 sequence-defined ASOs spanning six target families.

S1.3 Dataset versioning

Dataset construction proceeded through three deterministic preprocessing stages.

- Literature_master.csv: Raw literature aggregation containing all extracted efficacy measurements and associated metadata.
- Master_ASO_KD_Dataset.csv: Curated dataset containing one representative efficacy measurement for each ASO sequence.
- Master_ASO_KD_clean.csv: Final standardized dataset after label harmonization, metadata annotation, and target-family assignment.

Only the final standardized dataset was used for model training, calibration, and evaluation.

Experimentally generated DAZAP1 and FAM69C ASOs were maintained separately throughout preprocessing and were never incorporated into model training or hyperparameter optimization. Only experimentally measured ASOs satisfying predefined quality-control criteria were retained for external evaluation and family-specific calibration.

Table S1. Construction of the literature-derived ASO modeling dataset by target gene family.

Summary of antisense oligonucleotide (ASO) efficacy datasets by target gene family, showing raw aggregation counts, curated modeled counts, source domains, and primary knockdown assay modality.

Target gene / family	Initial literature aggregation (records)	Candidate records after standardization	Final modeled ASOs	Representative literature source(s)	Primary molecular endpoint
SOD1	79	7	6	Smith et al., 2006; McCampbell et al., 2018; Miller et al., 2020	mRNA knockdown
ATXN2	29	2	2	Becker et al., 2017; Scoles et al., 2017	mRNA knockdown
C9ORF72	7	9	5	Jiang et al., 2016; Donnelly et al., 2013	mRNA knockdown
FUS	30	1	1	Korben et al., 2022	Protein and mRNA knockdown
TGFBR2	4	–	0	Kuespert et al., 2020	mRNA knockdown

VCP	1	–	0	Pal et al., 2025	Protein knockdown
AR	–	3	3	Lieberman et al., 2014	mRNA knockdown
STMN2	–	5	5	Baughn et al., 2023	mRNA knockdown
UNC13A	–	1	0	Brown et al., 2022	mRNA knockdown
Total	150	28	22	–	–

Table Notes

- Initial literature aggregation denotes the complete collection of ASO efficacy records extracted from peer-reviewed publications and patent disclosures before dataset curation.
- Candidate records after standardization denotes literature-associated entries retained following sequence standardization, label harmonization, and metadata curation.
- Final modeled ASOs denotes sequence-defined ASOs retained for computational feature generation after exclusion of records lacking sufficient sequence information for feature computation.
- Literature sources represented in the modeled dataset identify the publications contributing sequence-defined ASOs retained in the final literature-derived modeling dataset.
- Initial aggregation counts and modeled ASO counts are not expected to correspond on a one-to-one basis because modeled datasets were assembled through deterministic curation, sequence standardization, and integration of sequence-defined literature sources.
- Experimentally generated DAZAP1 and FAM69C ASOs were maintained separately throughout preprocessing and are therefore not included in this table.

Supplementary Methods S2. Experimental validation

S2.1 Cell culture and ASO delivery

Experimental validation was performed using SH-SY5Y human neuroblastoma cells. ASOs were delivered by lipofection using Lipofectamine 3000 according to manufacturer protocols. Cells were incubated with ASO-lipid complexes prepared in OPTI-MEM before downstream RNA extraction.

S2.2 RNA isolation and cDNA synthesis

Total RNA was isolated from treated cells using standard RNA extraction procedures. RNA quality was assessed before reverse transcription. Complementary DNA (cDNA) was synthesized using ProtoScript II First Strand cDNA Synthesis Kit with oligo(dT) primers following manufacturer recommendations.

S2.3 Quantitative PCR

Transcript abundance was quantified using SYBR Green RT-qPCR with GAPDH as the internal reference gene. Technical replicates were performed for each reaction. Primer efficiency was evaluated using serial dilution standard curves generated from cDNA templates. Observed amplification efficiencies were approximately

- DAZAP1: 78%
- FAM69C: 94%
- GAPDH: 74%

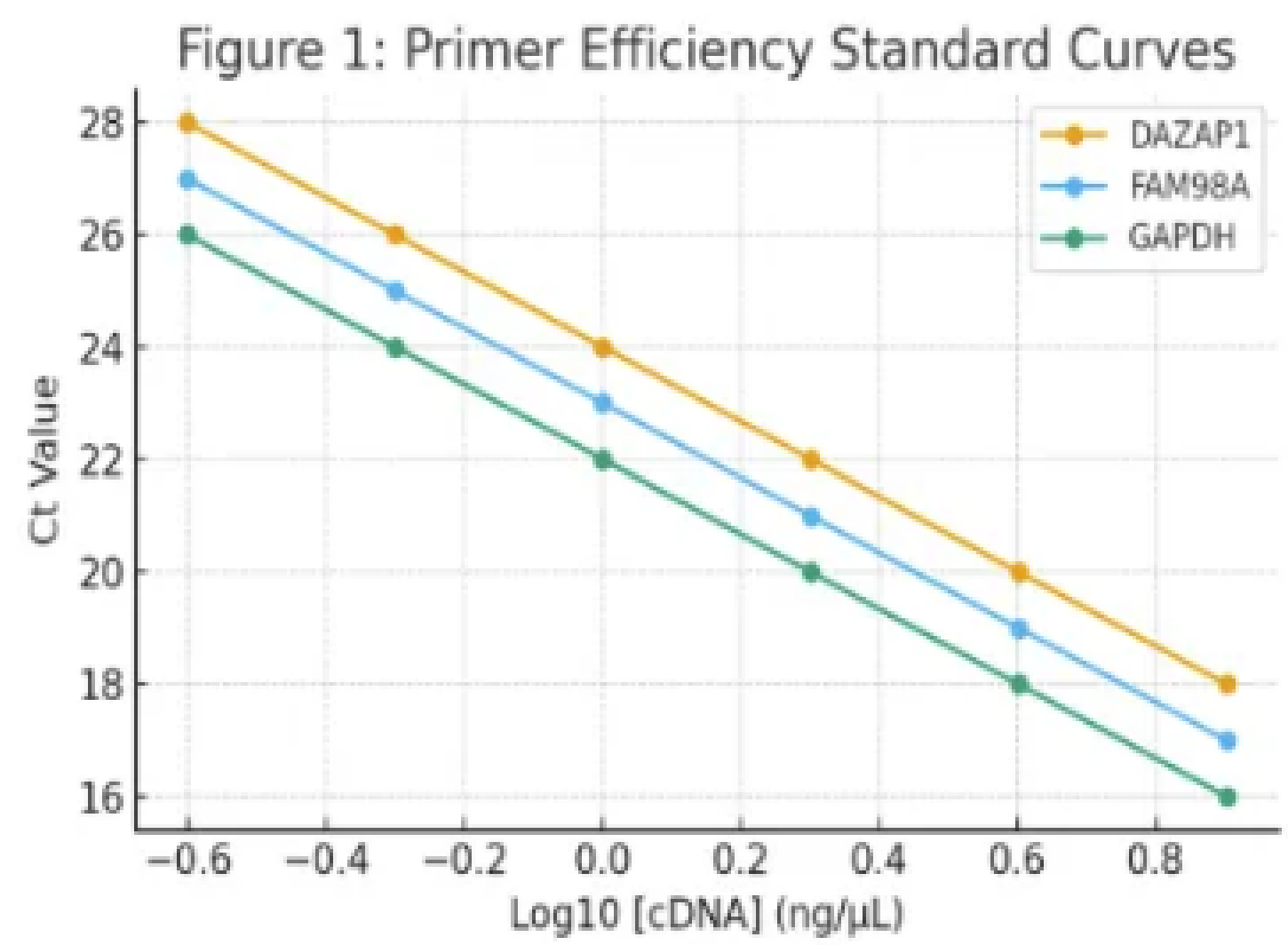

Supplementary Figure S1.

Primer efficiency validation. Shown to right.

Representative standard curves generated from serial dilution RT-qPCR assays for DAZAP1, FAM69C, and GAPDH. Amplification efficiencies were estimated from linear regression of Cq values against log-transformed template concentration.

S2.4 Knockdown quantification

DAZAP1 expression was quantified using the Pfaffl method to account for primer efficiencies deviating from ideal amplification. FAM69C expression was quantified using the ΔΔCq method because primer efficiency approached 100%. Percent knockdown was calculated relative to untreated controls following normalization to GAPDH.

S2.5 Quality control

Technical replicates were examined before downstream analysis. Measurements exhibiting failed amplification, abnormal melt curves, or excessive replicate variance were excluded. No-template controls were included throughout all RT-qPCR experiments to monitor contamination, and only quality-controlled measurements were incorporated into the computational evaluation dataset.

S2.6 Integration into model evaluation

Experimentally measured knockdown values for DAZAP1 and FAM69C were standardized using the same preprocessing pipeline as literature-derived measurements. These data were reserved exclusively for

out-of-distribution evaluation and one-shot calibration and were never used for model fitting or hyperparameter selection.

Table S2. Experimental ASOs and RT-qPCR metadata.

Target gene	ASO ID	Assay	Reference gene	Replicates
DAZAP1	WetLab_19	RT-qPCR	GAPDH	≥3
FAM69C	WetLab_8	RT-qPCR	GAPDH	≥3

Table S3. Raw observed and predicted knockdown values for experimentally evaluated ASOs underlying Figures 5 and 6.

ASO ID	Target family	Observed knockdown (%KD)	Predicted knockdown (%KD), uncalibrated	Predicted knockdown (%KD), one-shot calibrated	Role in one-shot calibration
19	DAZAP1	32.97	63.69	26.55	Evaluation ASO
20	DAZAP1	20.89	63.69	26.55	Independent evaluation ASO
21	DAZAP1	26.56	63.69	26.55	Evaluation ASO
22	DAZAP1	24.98	63.69	26.55	Evaluation ASO
**23**	**DAZAP1**	**26.55**	**63.69**	**26.55**	**Calibration reference**
24	DAZAP1	29.18	63.69	26.55	Evaluation ASO
**8**	**FAM69C**	**37.58**	**63.69**	**37.58**	**Calibration reference**
10	FAM69C	48.83	63.69	37.58	Evaluation ASO

Table notes

Observed transcript knockdown values were obtained from RT-qPCR experiments and were used exclusively for external model evaluation. Uncalibrated predictions correspond to the gradient boosting regression model before family-specific calibration. One-shot calibrated predictions incorporate the family-specific additive offset described in Section 2.6. ASO 23 (DAZAP1) and ASO 8 (FAM69C) served as the single calibration references used to estimate the family-specific offsets. All remaining ASOs were retained as independent evaluation observations. The values reported in this table correspond to the quantitative data underlying Figures 5 and 6.

Supplementary Methods S3. Label harmonization

All efficacy measurements were converted into a standardized transcript-level percent knockdown (%KD) representation before feature computation. Unless otherwise stated, %KD denotes reduction in target mRNA abundance relative to control.

S3.1 Standardization rules

Reported efficacy values were harmonized according to the following rules.

- Exact percentage values were retained unchanged.
- Residual expression values were converted as
  %KD = 100 − residual expression.
- Reported ranges were represented by the midpoint of the interval.
- Qualitative labels were mapped to fixed ordinal values:

Standardized values were constrained to the interval [0,100]. No weighting or uncertainty adjustment was applied beyond the information reported in the original publication.

S3.2 Provenance

Each standardized record retained metadata describing

- original reported value,
- label type,
- assay modality,
- source domain,
- target gene,
- literature reference.

These metadata enabled family-aware evaluation while preserving traceability to the original source.

Table S4. Examples of label harmonization.

Source type	Raw reported label	Label type	Standardized %KD	Notes
Literature	70–90%	Range	80	Interval midpoint
Literature	High	Qualitative	80	Ordinal mapping
Literature	Medium	Qualitative	55	Ordinal mapping
Literature	Low	Qualitative	20	Ordinal mapping
Literature	40% residual	Residual	60	100 − residual
Experimental	ΔΔCq-derived %	Exact	Computed	RT-qPCR

Table notes:
Numeric mappings for qualitative labels were selected to preserve ordering rather than imply quantitative precision.

Supplementary Figure S2. Distribution of standardized knockdown efficacy values.
Boxplots comparing standardized transcript-level knockdown measurements for literature-derived and experimentally generated ASOs following label harmonization are shown below.

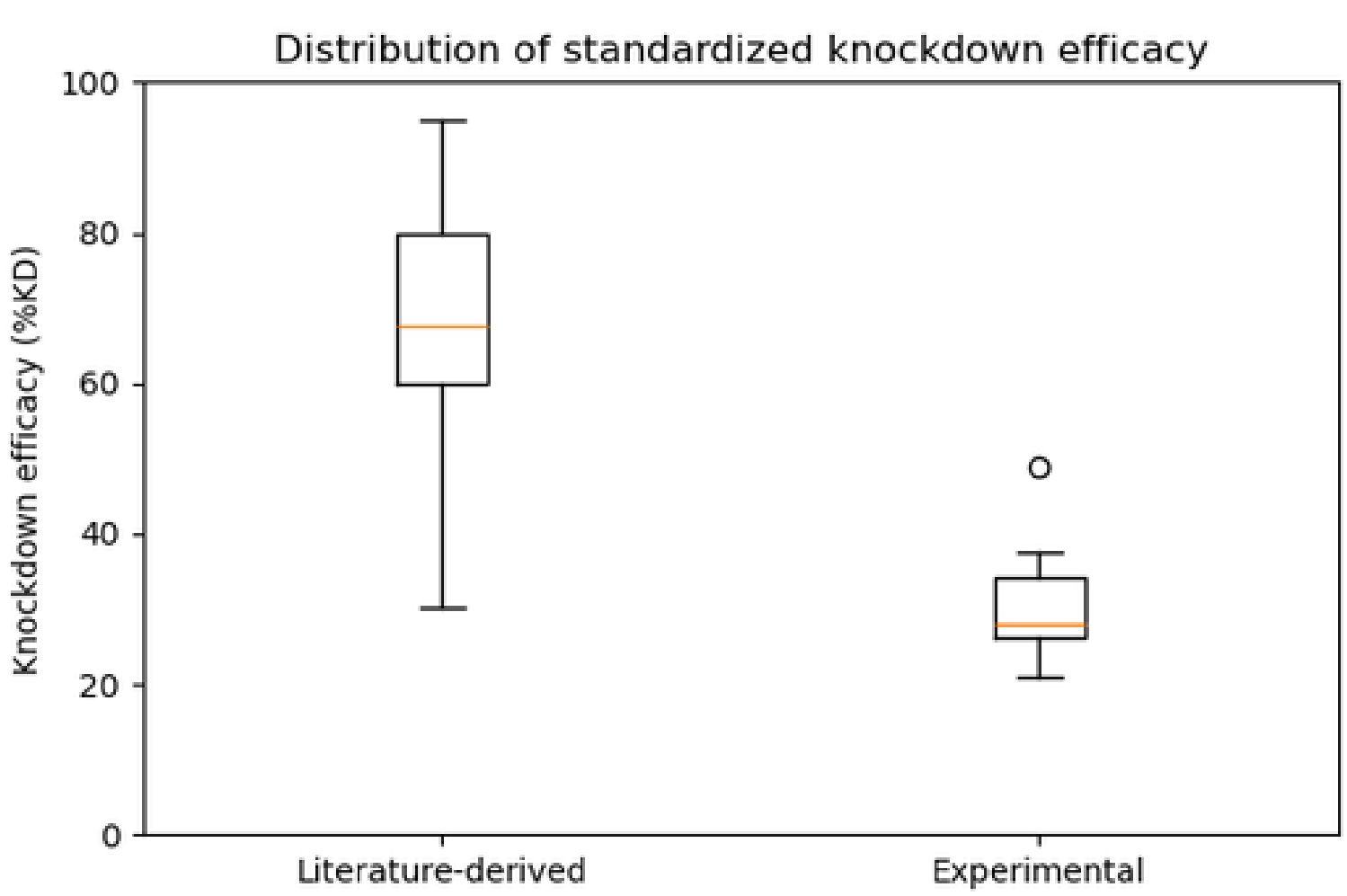

S4 Methods: Feature computation and biophysical parameters

All sequence-derived and biophysical features were computed deterministically from ASO sequences and target transcript annotations prior to model training. Feature computation was performed once per dataset version and cached to ensure consistent inputs across all modeling, calibration, and evaluation procedures.

S4.1 Sequence preprocessing

ASO sequences were represented in the 5′→3′ orientation and encoded using standard nucleotide alphabet notation. Sequences were required to meet minimum annotation criteria, including defined sequence length and target gene assignment. Sequences containing ambiguous nucleotides or unresolved bases were excluded prior to feature computation.

S4.2 Sequence composition features

Basic compositional features were computed directly from the ASO nucleotide sequence and included:

- total sequence length;
- overall GC content, defined as the fraction of guanine and cytosine bases;
- CpG density, defined as the number of CpG dinucleotides normalized by sequence length; and
- maximum homopolymer run length, defined as the longest contiguous stretch of identical nucleotides.

These features capture known influences of nucleotide composition on ASO binding affinity, stability, and off-target propensity.

S4.3 Gapmer regional features

For ASOs consistent with RNase H–active gapmer architectures, the sequence was partitioned into three regions: a 5′ flanking wing, a central DNA gap, and a 3′ flanking wing. Regional GC content was computed independently for each region to reflect the differential functional roles of the flanks and gap in RNase H recruitment and duplex stability. When explicit gapmer annotations were unavailable, canonical gapmer layouts reported in the source study were used. Sequences lacking sufficient information to support regional partitioning were excluded from gapmer-specific feature computation.

S4.4 Motif-based features

Low-order nucleotide motif features were computed to capture local sequence patterns. Dinucleotide and trinucleotide frequencies were calculated across the full ASO sequence and normalized by sequence length. These features provide a compact representation of local sequence context that has been associated with antisense efficacy and off-target interactions in prior studies.

S4.5 RNA–DNA hybridization thermodynamics

Thermodynamic estimates of duplex stability were computed using nearest-neighbor thermodynamic calculations implemented through the RNAduplex utility of the ViennaRNA package (Lorenz et al., 2011). The nearest-neighbor approach is based on established thermodynamic descriptions of nucleic acid duplex stability (Sugimoto et al., 1995; SantaLucia and Hicks, 2004). For each ASO–target pair, the resulting duplex free-energy estimate (ΔG_{duplex}) was used as a quantitative descriptor of relative ASO–target binding propensity.

S4.6 RNA secondary-structure accessibility

Target RNA accessibility was estimated using RNAplfold, an ensemble-based partition-function folding algorithm from the ViennaRNA package. For each ASO binding site, the probability that the contiguous target region was unpaired was used to derive accessibility descriptors, including an opening-energy interpretation and the logit-transformed unpaired probability. Accessibility estimates were derived from ensemble base-pairing probability distributions rather than a single minimum free-energy structure, thereby accounting for the dynamic structural ensemble of the target RNA (Bernhart et al., 2006; Lorenz et al., 2011; Wan et al., 2021).

S4.7 Combined effective binding proxy

To integrate hybridization strength and target accessibility, a combined effective binding proxy was computed conceptually as the difference between duplex formation free energy and the RNA structural accessibility penalty. This formulation reflects the requirement that productive RNase H–mediated knockdown depends on both stable hybridization and accessibility of the target site. This combined quantity was evaluated as a derived biophysical feature during model development. When accessibility estimates could not be computed due to sequence length or structural constraints, models were trained using sequence-only and thermodynamic features to ensure consistent treatment across datasets.

S4.8 Formal definitions of sequence-derived features

Let $S = (S_1, \ldots, S_n)$ denote an ASO nucleotide sequence of length n, with $S_i \in \{A, C, G, T\}$.

<u>Motif frequencies</u>

For each dinucleotide $xy \in \{A, C, G, T\}^2$, the normalized dinucleotide frequency d_{xy} is defined as

$$d_{xy} = |\{ i : S_i S_{i+1} = xy \}| / (n - 1), \qquad \text{with } d_{xy} = 0 \text{ when } n < 2.$$

When included, trinucleotide frequencies are computed analogously. For each trinucleotide $xyz \in \{A, C, G, T\}^3$,

$$tri_{xyz} = |\{ i : S_i S_{i+1} S_{i+2} = xyz \}| / (n - 2), \qquad \text{with } tri_{xyz} = 0 \text{ when } n < 3.$$

These motif frequencies provide a compact representation of local sequence context and enable enrichment analyses across efficacy strata.

<u>Gapmer regional GC content (5–10–5 approximation)</u>

For ASO sequences with $n \geq 20$, a canonical 5–10–5 gapmer architecture is approximated. The sequence is partitioned into a 5′ wing ($S_1 \ldots S_5$), a central DNA gap ($S_6 \ldots S_{15}$), and a 3′ wing ($S_{□-4} \ldots S_{□}$).

Regional GC fractions are computed as

$$GC_{left} = (|\{G \text{ in } S_1 \ldots S_5\}| + |\{C \text{ in } S_1 \ldots S_5\}|) / 5,$$

$$GC_{gap} = (|\{G \text{ in } S_6 \ldots S_{15}\}| + |\{C \text{ in } S_6 \ldots S_{15}\}|) / 10,$$

$$GC_{right} = (|\{G \text{ in } S_{□-4} \ldots S_{□}\}| + |\{C \text{ in } S_{□-4} \ldots S_{□}\}|) / 5.$$

<u>Thermodynamic and accessibility proxy</u>

When available, RNA–DNA duplex hybridization free energy (ΔG_{duplex}) and RNA structural accessibility penalties (ensemble defect, ED) are combined into an effective binding proxy defined as

$$\Delta G_{eff} = \Delta G_{duplex} - ED.$$

This formulation reflects the requirement that productive RNase H–mediated knockdown depends on both stable hybridization and accessibility of the target RNA site.

S4.9 Feature standardization and caching

All features were standardized using statistics computed on the training data only and applied unchanged to held-out and out-of-distribution datasets. Feature matrices were saved as versioned artifacts and reused across all model variants to prevent inadvertent information leakage and ensure reproducibility. Feature computation parameters were fixed for the duration of the study. Accordingly, ΔG_{eff} was evaluated as a derived biophysical descriptor during model development.

Table S5. Summary of computed feature categories

Feature category	Feature examples	Description
Sequence composition	Sequence length, GC content, CpG density	Global nucleotide composition properties of the ASO sequence that influence binding propensity and stability.
Gapmer regional composition	GC5′, GCgap, GC3′	Region-specific GC fractions computed over canonical gapmer subregions to reflect differential roles in RNase H recruitment and duplex stability.
Motif composition (k-mer)	5-mer composition components, 6-mer	Principal components summarizing correlated k-mer frequency patterns,

	composition components	capturing global sequence architecture and motif enrichment.
Thermodynamics	ΔG_{duplex}, Tm, mismatch penalties	RNA–DNA hybridization energetics estimated using nearest-neighbor models, reflecting intrinsic duplex stability.
Target accessibility	Ensemble defect (ED)	Target accessibility: $p_{unpaired,site}$, logit_punp, and opening-energy interpretation (ED) Site-level accessibility descriptors derived from RNAplfold ensemble unpaired probabilities
Combined binding proxy	$\Delta G_{eff} = \Delta G_{duplex} - ED$	Derived biophysical descriptor integrating hybridization strength and target accessibility, evaluated during model development
Seed-region features	Seed k-mer counts, seed fraction	Local motif statistics computed over defined seed regions to capture short-range sequence effects.
Chemical architecture constraints	Gapmer architecture, length constraints	Structural and design constraints applied to RNase H–active ASOs, used for feature computation and inclusion filtering but not as predictive labels.

Table notes:
This table summarizes feature categories; the complete list of computed features is provided in Supplementary Table S6.

S4.10 Software tools and versions

All feature computation was performed using fixed, versioned software tools to ensure reproducibility across model variants and evaluation stages.

- Sequence preprocessing and motif features
  Implemented in Python (v3.10) using custom scripts built on pandas (v1.5+) and numpy (v1.23+).

- RNA secondary-structure accessibility
  RNA accessibility estimates were computed using ViennaRNA (v2.4.7), specifically the RNAplfold utility, which computes base-pairing probabilities across a sliding window and reports ensemble-based accessibility metrics (Lorenz et al., 2011).
- RNA–DNA duplex thermodynamics
  RNA–DNA duplex stability estimates were computed using the RNAduplex utility from the ViennaRNA package (v2.4.7), which implements nearest-neighbor thermodynamic calculations for duplex free-energy estimation. The nearest-neighbor approach is based on established thermodynamic descriptions of nucleic acid duplex stability (Sugimoto et al., 1995; SantaLucia and Hicks, 2004). RNA secondary-structure accessibility was computed using RNAplfold (ViennaRNA v2.4.7), which estimates local RNA accessibility using ensemble partition-function folding (Lorenz et al., 2011; Bernhart et al., 2006).

No learning algorithms or feature definitions were modified after feature cache generation. All downstream modeling steps consumed identical cached feature matrices.

S4.11 RNA accessibility windowing and parameters

RNA secondary-structure accessibility was computed using RNAplfold from the ViennaRNA package (v2.4.7), which estimates local unpaired probabilities using ensemble partition-function folding. RNAplfold was run with a local folding-window length of 120 nucleotides (-W 120), a maximum unpaired-region length of 20 nucleotides (-u 20), and the --noLP option to exclude isolated base pairs. RNAplfold reports the probability that a contiguous region of length l is unpaired at each transcript position. For each mapped 20-nucleotide ASO target site, the site-level unpaired probability was extracted as

$$p_{unpaired,site} = P_u(i, l=20),$$

where i denotes the mapped start position and l is the length of the unpaired region.

For interpretation in energetic terms, the accessibility or opening-energy penalty was defined as

$$ED(i) = -RT \ln p_{unpaired,\ site},$$

where R is the gas constant and T is the absolute temperature. In the implemented feature pipeline, the site-level probability was additionally transformed using

$$logit\_punp = \ln\left(p_{unpaired,site} / (1 - p_{unpaired,site})\right),$$

and logit_punp was retained as the numerical accessibility descriptor used in the publication feature snapshot.

S4.12 RNA–DNA duplex free-energy calculation

Duplex free-energy estimates (ΔG_{duplex}) were obtained using RNAduplex under standard physiological conditions and used as relative descriptors of ASO–target hybrid stability.

- Strand orientation: RNA (target) : DNA (ASO)
- Parameterization: nearest-neighbor stacking energies for RNA–DNA hybrids
- Salt and temperature assumptions: standard conditions consistent with reported parameter sets (37 °C)

The reported ΔG_{duplex} represents the total standard-state Gibbs free energy of duplex formation for the full ASO–RNA complement, rather than a per-base average.

S4.13 Handling of missing accessibility estimates

Accessibility estimates could not be computed for all ASO–target pairs due to limitations such as:

- incomplete transcript context,
- ambiguous binding-site localization,
- or incompatibility with RNAplfold windowing constraints.

To ensure consistent treatment across datasets:

- No imputation was performed for missing accessibility values.
- When accessibility estimates were unavailable, the combined effective binding proxy ΔG_{eff} was not computed.
- Models trained on feature sets requiring accessibility were restricted to ASOs with complete thermodynamic and accessibility coverage.
- Parallel models using sequence-only or sequence + thermodynamic features were trained to maintain parity across literature and experimental datasets.

This strategy avoids introducing artificial signal through imputation while preserving maximal data inclusion in lower-order feature regimes.

S4.14 Feature scaling and normalization

All numeric features were standardized using z-score normalization:

$$x' = (x - \mu_{train}) / \sigma_{train}$$

where μ_{train} and σ_{train} were computed exclusively on the training set within each evaluation regime.

The same normalization parameters were applied unchanged to:

- validation folds,
- held-out families,
- and out-of-distribution experimental datasets.

This prevents feature leakage and ensures that performance differences arise solely from modeling choices rather than preprocessing artifacts.

S4.15 Feature caching and version control

Feature computation was executed once per dataset version and saved as a versioned feature artifact keyed by:

- ASO identifier,
- target gene,
- sequence hash,
- feature-set version tag.

All modeling, calibration, and evaluation experiments consumed these cached artifacts without recomputation. When any upstream rule changed (e.g., label harmonization logic or accessibility parameters), the feature version tag was incremented and the cache rebuilt in full.

This approach ensures strict reproducibility and enables fair comparison across feature configurations.

Table S6. Complete list of computed features used in model development.

Feature category	Feature name	Description
Composition	seq_len	ASO sequence length (nucleotides)
Composition	$GC_{content}$	Fraction of G and C bases across the full ASO
Composition	$CpG_{density}$	Normalized frequency of CpG dinucleotides
Composition	max_homopolymer	Length of longest contiguous nucleotide run
Gapmer architecture	GC_{left}	GC fraction in 5′ wing (positions 1–5)
Gapmer architecture	GC_{gap}	GC fraction in central DNA gap (positions 6–15)
Gapmer architecture	GC_{right}	GC fraction in 3′ wing (last 5 bases)
Motifs	di_*	Normalized dinucleotide frequencies (16 features)
Motifs	tri_*	Normalized trinucleotide frequencies (64 features; optional)
Thermodynamics	ΔG_{duplex}	RNA–DNA duplex hybridization free energy
Accessibility	ED	Mean ensemble defect (opening energy)
Combined	ΔG_{eff}	Effective binding proxy: ΔG_{duplex} - ED

Table notes:

Feature names correspond to cached feature matrices used for model training and evaluation. Wildcards (e.g., di_*, tri_*) denote families of related features. Highly correlated features were compressed using principal component analysis prior to model training.

Supplementary Methods S5. Model implementation

S5.1 Prediction task

ASO efficacy prediction was formulated as a supervised regression problem, with standardized transcript-level knockdown (%KD) used as the response variable. The primary model was a gradient-boosted regression tree, chosen for its ability to capture nonlinear feature interactions while remaining robust in small-sample settings.

S5.2 Model configuration

The gradient boosting models employed shallow regression trees, squared-error loss, conservative learning rates, and fixed random seeds. Hyperparameter search was deliberately restricted to biologically plausible ranges to reduce the risk of overfitting. The final configuration is summarized in Table S6.

S5.3 Input features

Models were trained exclusively on the precomputed feature matrices described in Supplementary Methods S4. Categorical metadata, including target gene, assay type, and source domain, were not used as model inputs and were reserved for dataset stratification and downstream analyses. No missing-value imputation was performed.

S5.4 Software

Model development was conducted using Python 3.10 with the following libraries: scikit-learn (version 1.2 or higher), NumPy (version 1.23 or higher), and pandas (version 1.5 or higher). All analyses were executed with fixed random seeds to ensure reproducibility.

S5.5 One-shot calibration

Family-specific calibration was implemented as a post hoc additive correction: $\hat{y}_i^{cal} = \hat{y} + (y_c - \hat{y}_c)]$

where $\hat{y}_c$ denotes the experimentally measured knockdown of the calibration ASO. Calibration was applied only during evaluation and was not used during model training.

S5.6 Reproducibility

Model configuration, feature versions, random seeds, and preprocessing settings were fixed prior to evaluation. No adaptive learning, online updates, or post hoc hyperparameter optimization were performed after feature generation.

Table S7. Final model configuration.

Parameter	Value	Notes
Model type	Gradient-boosted regression trees	Shallow trees
Loss function	Squared error	Optimized during training
Max tree depth	3–4	Selected for stability
Learning rate	~0.01	Conservative shrinkage
Number of estimators	~1500–2000	Early stopping not used
Min samples per leaf	1	Small-n regime
Feature scaling	Z-score (train-only)	See S4.14
Random seed	Fixed	Reproducible

Table notes:

Parameters shown correspond to the final configuration used for all reported results. Hyperparameter values were selected based on stability across family-aware validation splits and were fixed prior to out-of-distribution evaluation. No hyperparameters were tuned using held-out or experimental target families. Feature scaling and preprocessing were applied using statistics computed on training data only.

Table S8. Model evaluation regimes and sample sizes.

Target family	Data source	Sample size (n)	Evaluation regime
SOD1	Literature	6	Family-aware k-fold CV
ATXN2	Literature	2	LOOCV
C9ORF72	Literature	5	Family-aware k-fold CV
FUS	Literature	1	Held out (descriptive only)

DAZAP1	Experimental	6	OOD evaluation + optional calibration
FAM69C	Experimental	2	OOD evaluation + optional calibration

Table notes:

Sample sizes reflect the curated, model-ready dataset retaining one representative measurement per ASO sequence. Evaluation regimes were selected based on per-family sample availability. Experimental target families were excluded from all training and tuning procedures and were used exclusively for out-of-distribution evaluation.

Supplementary Results S7. Residual analysis and uncertainty after family-specific calibration

To further characterize model performance following one-shot family-specific calibration, we examined prediction residuals and uncertainty estimates using the independently generated experimental ASOs. These analyses complement the quantitative performance metrics presented in the main manuscript by evaluating whether calibration removes systematic bias while preserving stable prediction behavior under small-sample conditions.

Residual distributions for each target family are shown in Supplementary Figure S7A. Following calibration, residuals for DAZAP1 were centered near zero with relatively limited dispersion, indicating effective correction of family-level prediction bias. Residuals for FAM69C showed greater variability, which is expected given the very small number of experimentally measured ASOs available for this target ($n = 2$). Although uncertainty differed between families, calibration consistently removed the systematic directional bias observed before correction.

Residuals plotted against observed knockdown efficacy (Supplementary Figure S7B) showed no clear relationship between residual magnitude and observed transcript-level knockdown after calibration. The absence of an obvious trend suggests that the additive correction improves overall prediction scale without introducing systematic error across the range of observed knockdown values.

Prediction uncertainty was quantified using nonparametric bootstrap resampling of the experimental dataset. Bootstrap distributions of mean absolute error (MAE) are shown in Supplementary Figure S7C. As expected, bootstrap estimates for DAZAP1 were relatively concentrated, whereas estimates for FAM69C exhibited substantially broader distributions because of the limited sample size. This increased variability should therefore be interpreted as reflecting uncertainty in performance estimation rather than reduced model reliability.

Collectively, these analyses support the conclusions presented in the main manuscript. One-shot calibration effectively removes family-specific prediction bias while maintaining stable behavior across experimentally

measured targets, although confidence in family-specific performance estimates remains dependent on the availability of experimental observations.

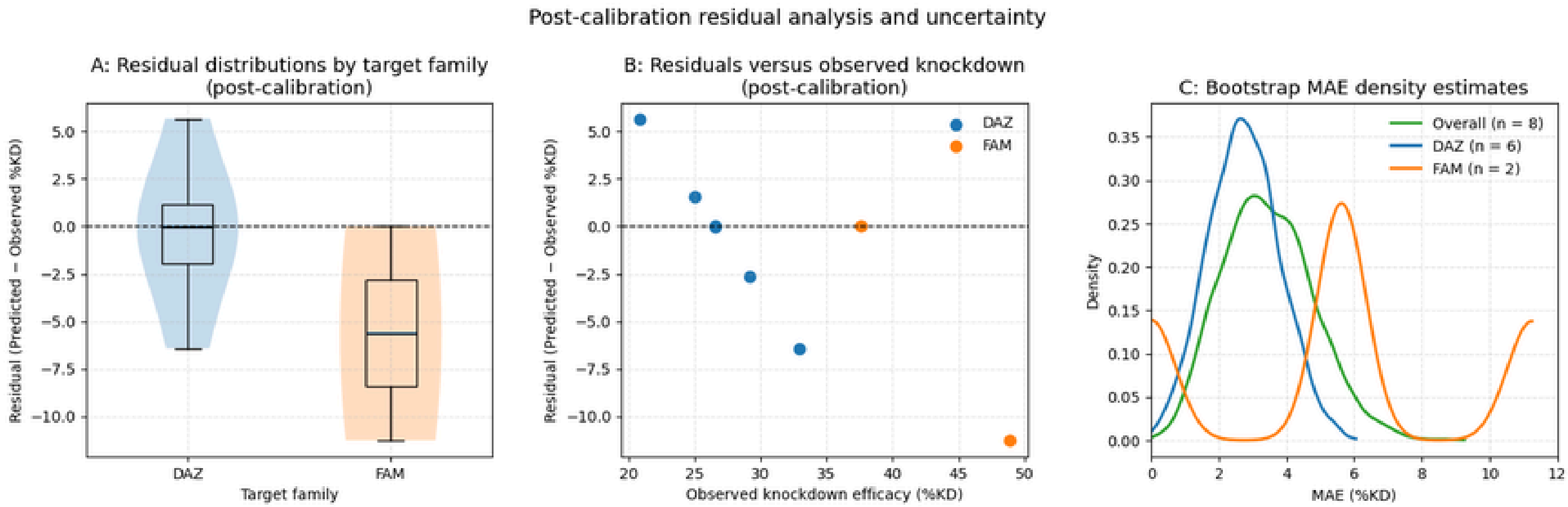

Supplementary Figure S7. Post-calibration residual analysis and uncertainty.

(A) Distribution of prediction residuals (predicted − observed %KD) following one-shot calibration for each experimental target family. (B) Residuals plotted against observed transcript-level knockdown, demonstrating the absence of systematic dependence of prediction error on knockdown magnitude following calibration. (C) Bootstrap distributions of mean absolute error (MAE) for the overall experimental dataset and individual target families. The broader distribution observed for FAM69C reflects the limited number of experimentally measured ASOs rather than systematic model failure.

Supplementary Results S8. Leave-one-family-out validation of one-shot calibration

To evaluate the one-shot calibration strategy independently of the limited experimental validation set, we performed repeated leave-one-family-out (LOFO) simulations using the literature-derived ASO dataset. In each simulation, all ASOs belonging to a single target family were withheld during model training. Predictions were first generated without calibration, after which one ASO from the held-out family was randomly designated as the calibration reference. The resulting additive offset was then applied uniformly to the remaining ASOs within that family, and prediction accuracy before and after calibration was compared. This procedure was repeated 1,000 times for every eligible target family to characterize variability arising from calibrator selection.

The simulation results demonstrate that calibration performance depends strongly on the characteristics of the target family (Supplementary Table S8). Families exhibiting a consistent family-level prediction offset generally showed substantial reductions in mean absolute error following calibration, indicating that a single experimentally measured reference ASO can effectively correct systematic prediction bias. In contrast, families displaying greater within-family heterogeneity showed smaller improvements or, in some cases, slight reductions in performance, suggesting that a uniform additive correction cannot completely account for more complex sources of variability.

Importantly, these simulations indicate that one-shot calibration does not artificially improve prediction accuracy across all datasets. Instead, improvements occur primarily when the assumptions underlying the calibration procedure are satisfied, namely that prediction error is dominated by an approximately constant family-specific offset. This behavior supports the use of one-shot calibration as a conservative correction strategy rather than a replacement for additional experimental measurements.

Families containing very few ASOs (n ≤ 3) were retained for completeness but were not considered informative for evaluating calibration robustness because the limited number of possible calibration configurations results in inherently unstable performance estimates.

Together with the independent experimental validation presented in the main manuscript, these simulations demonstrate that one-shot calibration can substantially improve absolute prediction accuracy under realistic small-data conditions while appropriately reflecting family-specific variability.

Supplementary Table S9. Summary of leave-one-family-out one-shot calibration simulations.

Family	n (Family)	MAE (uncalibrated)	ΔMAE Mean	ΔMAE Median	ΔMAE SD	ΔMAE 95% CI Low	ΔMAE 95% CI High	Fraction improved
DAZ	6	0.246	0.197	0.204	0.019	0.163	0.213	1
C9ORF72	5	0.108	-0.003	0.045	0.057	-0.080	0.045	0.581
STMN2	5	0.230	-0.011	0.008	0.067	-0.092	0.058	0.595
SOD1	6	0.163	-0.028	-0.022	0.040	-0.078	0.023	0.332
ATXN2	2	0.095	0.075	0.085	0.010	0.065	0.085	1
FAM	2	0.106	-0.005	0.050	0.056	-0.063	0.050	0.518
AR	3	0.21688	-0.017523	0	0.023631	-0.04936	0	0

For each target family, all ASOs were withheld during model training and one ASO was randomly selected as the calibration reference in each of 1,000 repeated simulations. Reported statistics summarize changes in mean absolute error following calibration, together with empirical 95% confidence intervals and the proportion of simulations in which calibration reduced prediction error. Families containing three or fewer ASOs are included for completeness but were not used to evaluate calibration robustness because of the limited number of possible calibration configurations.
